# Neutrophil Recruitment via Hepatocyte IL-1α Drives NETs-Mediated AIM2 Hepatocyte Apoptosis in Alcohol-associated steatohepatitis

**DOI:** 10.7150/ijbs.121255

**Published:** 2025-09-03

**Authors:** Yuan Zhang, Xueteng Meng, Yan Ding, Jinmao Yu, Yunyun Wan, Zhiying Yang, Zheyu Han, Qian Zhu, Rui Feng, Jun Li, Cheng Huang, Taotao Ma

**Affiliations:** Inflammation and Immune Mediated Diseases Laboratory of Anhui Province; Anhui Institute of Innovative Drugs; School of Pharmacy, Anhui Medical University, Hefei 230032, Anhui, China.

**Keywords:** alcohol-associated steatohepatitis, neutrophil extracellular traps, IL 1α, AIM2, apoptosis

## Abstract

Alcohol-associated steatohepatitis (ASH) represents a critical stage in the progression of Alcohol-associated liver disease (ALD), characterized by extensive hepatocellular steatosis, immune cell infiltration, and a poor therapeutic response. Neutrophils play a central role in the inflammatory landscape of ASH, with their hepatic accumulation strongly correlating with disease severity. Although studies have demonstrated that neutrophil depletion attenuates liver injury, the precise mechanisms underlying neutrophil-mediated hepatocellular damage remain poorly defined. Neutrophil extracellular traps (NETs), web-like DNA structures released during NETosis, have emerged as key effectors in sterile inflammation and may exacerbate liver injury beyond their antimicrobial functions. In this study, we employed the Binge-Gao mouse model to explore the involvement of NETs in ethanol-induced liver injury. Our findings revealed that ethanol exposure led to significant hepatic neutrophil infiltration and NET formation. Stressed hepatocytes released damage-associated molecular patterns (DAMPs), particularly interleukin-1 alpha (IL-1α), which activated Toll-like receptor 9 (TLR9) on neutrophils, thereby enhancing NET generation. NET components subsequently activated the cytosolic DNA sensor AIM2 (absent in melanoma 2) in hepatocytes, triggering apoptosis. This cascade illustrates a previously unrecognized immune axis linking ethanol-damaged hepatocytes, NET-producing neutrophils, and DNA-sensing death pathways.

## Introduction

The liver serves as the primary organ of ethanol metabolism. The cytotoxicity of ethanol and its metabolites can induce liver injury. Chronic excessive alcohol consumption is the leading cause of Alcohol-associated liver disease (ALD) 1. Based on pathological progression, ALD can be classified into alcoholic fatty liver, Alcohol-associated steatohepatitis (ASH), alcoholic cirrhosis, and hepatocellular carcinoma 2. ASH represents a critical, treatment-refractory stage during disease progression. The hallmark pathological features of ASH include significant hepatocellular steatosis and immune cell infiltration 3. Studies demonstrate that mild inflammation accelerates hepatic fibrogenesis 4, while severe inflammation can precipitate liver failure or death 5. Although systemic immunosuppressants, such as corticosteroids, can suppress hepatic inflammation, this therapeutic approach incurs an increased risk of systemic pathogen infections 6. Therefore, shifting the therapeutic focus towards maintaining inflammatory homeostasis, rather than solely pursuing immunosuppression, is now considered the more rational approach.

Clinical studies have demonstrated that hepatic infiltration of neutrophils is a prominent pathological feature in patients with ASH, and the extent of neutrophil accumulation correlates positively with disease severity 7. Experimental depletion of neutrophils using anti-Ly6G antibodies has been shown to markedly alleviate liver injury 8, highlighting the pathogenic role of neutrophils in the progression of ASH. One of the key effector mechanisms through which neutrophils exert their immunological function is the formation of neutrophil extracellular traps (NETs) 9. While NETs are essential for capturing and neutralizing pathogens 10, their uncontrolled release during sterile inflammation has been shown to amplify tissue damage 11. For example, NETs have been shown to aggravate hepatocellular injury during hepatic ischemia-reperfusion 12. Recent work by our group has revealed that, in drug-induced liver injury, NETs can activate the hepatocellular cytosolic DNA sensor Aim2^13^, thereby triggering programmed cell death. Despite these findings, the functional significance and mechanistic pathways of NETs in ethanol-induced liver injury remain largely undefined.

In various sterile inflammatory diseases, the initiation of inflammation has been closely linked to intercellular communication between parenchymal cells and immune cells 14. Huang et al. demonstrated that parenchymal cells act as sensors of organ homeostasis by detecting endogenous danger signals such as changes in osmotic pressure, nutrient deprivation, and the accumulation of toxic metabolites 15. Upon injury, these cells release damage-associated molecular patterns (DAMPs) into the circulation. Immune cells subsequently recognize these DAMPs through pattern recognition receptors (PRRs), migrate to the injury site, and become activated to initiate inflammation 16. Among these, the interleukin (IL) family has emerged as a key group of DAMPs produced by parenchymal cells. For instance, hepatocytes under lipid metabolic stress have been shown to secrete IL-11, which activates hepatic stellate cells and promotes fibrosis 17. Similarly, in models of kidney injury, tubular epithelial cells release IL-19 to convey aristolochic acid-induced cytotoxic signals to neutrophils 18. These findings suggest that parenchymal cells play an active role in the propagation of sterile inflammation. However, in the context of alcohol-induced liver injury, the mechanisms by which hepatocytes sense alcohol-related metabolic stress and communicate these signals to immune cells remain poorly understood. Further investigation is needed to elucidate the communication between hepatocyte and immune cells before an outbreak of inflammation in ASH.

In this study, we used the Binge-Gao model to demonstrate that neutrophils induce hepatocyte apoptosis via NET formation in ASH mice. Further mechanistic studies showed that ethanol-stressed hepatocytes release DAMPs, particularly interleukin-1 alpha (IL-1α), which then upregulate Toll-like receptor 9 (TLR9) on neutrophils. This signaling pathway promotes neutrophil recruitment to the liver and enhances NET formation. Ultimately, NET components activate the cytosolic double-stranded DNA sensor absent in melanoma 2 (AIM2) in hepatocytes, triggering programmed cell death. These findings reveal a previously unrecognized immune interaction between neutrophils and hepatocytes, offering new insights into ASH pathogenesis.

## Materials and Methods

### Animals and treatment

All experimental procedures in this study were approved by the Animal Experimentation Ethics Committee from Anhui Medical University. Wild-type (WT) C57BL/6J mice and AIM2 knockout mice (on a C57BL/6J background) were obtained from the GemPharmatech (Nanjing, China). All experiments described in this report used male mice aged 8 - 10 weeks. Mice were housed in a temperature (23 ± 2 ℃) and humidity (40 ± 5%) controlled specific pathogen-free facility with a 12 h/12 h dark/light photocycle. For the chronic-plus-binge NIAAA alcohol-associated hepatitis mouse model, mice received Lieber DeCarli for 10 days and additional binge alcohol on the last day as previously described 19. The liver tissues and blood from these mice were collected after the last alcohol gavage for further analysis.

### Adeno-associated virus 8 (AAV8) injection

AAV8 vectors were constructed and packaged by Hanbio Biotechnology Co., Ltd. (Shanghai, China). For overexpression studies, mice were intravenously injected with an AAV8 vector encoding the full-length mouse AIM2 gene (AAV8-AIM2). To knock down IL-1α, mice received an AAV8 vector expressing a short hairpin RNA targeting the mouse IL-1α gene (AAV8-shIL-1α). Each mouse was injected via the lateral tail vein with 200 μL of viral solution containing 2.5 × 10¹¹ vector genomes.

### DNase I, IL-1α and TLR9 inhibitor treatment

To evaluate the effect of NET-DNA and IL-1α signaling, mice were treated with DNase I (10 U in 100 μL 0.9% NaCl, Intraperitoneal injection (i.p.); Cat. No. EN0521, Thermo Fisher Scientific/Fermentas, USA) or the TLR9 inhibitor E6446 (20 mg/kg, Oral administration (p.o.); Cat. No. S6719, Selleck, China) at 24 and 15 hours prior to tissue harvest. For IL-1α administration, mice received recombinant mouse IL-1α (10 μg/kg, diluted in 0.9% NaCl; intrahepatic injection) at 15 hours before euthanasia to mimic local inflammatory signaling. IL-1α was obtained from MCE (Cat. No. HY-P7072, MedChemExpress, China).

### Oil red O staining

The frozen liver tissues were sliced into 10 μm-thick sections and then stained with Oil Red O working solution at RT. Subsequently, the sections were washed 3 times with PBS and counterstained with hematoxylin. Images were acquired using an automated digital slide scanner (Pannoramic MIDI, 3DHISTECH, Budapest, Hungary).

### Hematoxylin-eosin (H&E) stainning

To assess pathological changes in liver tissues, liver tissues were harvested, fixed in 4% paraformaldehyde, embedded in paraffin, and cut into sections (5 μm thick). The sections were stained with hematoxylin and eosin according to the H&E staining kit (Cat. No. C0105S; Beyotime, Shanghai, China) instructions. Images were acquired using an automated digital slide scanner (Pannoramic MIDI, 3DHISTECH, Budapest, Hungary).

### Immunohistochemical (IHC) and immunofluorescence (IF) staining

Liver-infiltrating neutrophils were detected by IHC staining using a primary antibody against myeloperoxidase (MPO) (Cat. No. ab208670; Abcam, USA). Tissue sections were incubated with the anti-MPO antibody at the dilution of 1/1000 overnight at 4 °C, followed by incubation with a suitable secondary antibody and visualization using DAB substrate. Images were acquired using an automated digital slide scanner (Pannoramic MIDI, 3DHISTECH, Budapest, Hungary).

To detect NETs in liver tissue, paraffin-embedded sections were first deparaffinized, rehydrated, and subjected to antigen retrieval. After blocking with 5% normal goat serum for 1 h at room temperature, sections were incubated overnight at 4 °C with a rabbit monoclonal antibody against CitH3 (1:100; Cat. No. EPR20358-120; Abcam, USA). The next day, sections were washed and incubated with Alexa Fluor® 488-conjugated goat anti-rabbit IgG(H+L) (1:100; Cat. No. AF-0511; ASGB-Bio, Beijing, China) for 1 h at room temperature. To minimize cross-reactivity from using two rabbit primary antibodies, sections were blocked again with 5% goat serum, followed by incubation with a second rabbit monoclonal antibody against MPO (1:100; Cat. No. ab208670; Abcam, USA) for 2 h at room temperature. After washing, sections were incubated with Alexa Fluor® 594-conjugated goat anti-rabbit IgG (H+L) (1:100; Cat. No. AF-0516; ASGB-Bio, Beijing, China) for 1 h. Nuclear counterstaining was performed with DAPI (G1407; Servicebio, Wuhan, China) for 10 min. Images were captured using a confocal laser scanning microscope. Images were acquired using a digital slide scanner (Pannoramic MIDI, 3DHISTECH, Budapest, Hungary).

### TUNEL assay

Apoptotic cells in liver tissue were detected using a TUNEL apoptosis detection kit (colorimetric method) (C1091; Beyotime, Shanghai, China), following the manufacturer's instructions. Briefly, paraffin-embedded liver sections were deparaffinized, rehydrated, and treated with proteinase K for antigen retrieval. After washing with PBS, sections were incubated with the TUNEL reaction mixture at 37 °C for 1 h. Subsequently, sections were incubated with horseradish peroxidase (HRP)-conjugated streptavidin, followed by DAB substrate development to visualize apoptotic nuclei as brown signals. Hematoxylin was used for counterstaining. Images were acquired using an automated digital slide scanner (Pannoramic MIDI, 3DHISTECH, Budapest, Hungary). Semi-quantitative analysis of TUNEL-DAB staining was performed using ImageJ (NIH, Bethesda, MD, USA). DAB-positive areas were segmented by color thresholding, and the percentage of TUNEL-positive area relative to total tissue area was calculated.

### Analysis of serum parameters

Serum levels of alanine aminotransferase (ALT) and aspartate aminotransferase (AST) were quantified using commercial activity assay kits (ALT: P/N 105-000442-00; AST: P/N 105-000443-00; Mindray, Shenzhen, China) according to the manufacturer's instructions. Myeloperoxidase (MPO) and double-stranded DNA (dsDNA) concentrations in serum were measured using an MPO ELISA kit (Cat. No. MU30238; Bioswamp, Wuhan, China) and a dsDNA High Sensitivity Assay Kit (Cat. No. 12640ES76; Yeasen, Shanghai, China), respectively. Serum interleukin-1 alpha (IL-1α) levels were determined using a specific ELISA kit (Cat. No. MU30598; Bioswamp, Wuhan, China).

### Flow cytometry analysis of hepatic nonparenchymal cells (NPCs)

Mouse livers were harvested and immediately transferred into ice-cold PBS. After removal of the gallbladder and visible blood vessels, liver tissues were cut into small pieces and mechanically dissociated using a 70-μm cell strainer (Cat. No. 352350; Corning, USA) placed over a 50 mL conical tube. A sterile plunger from a 5 mL syringe was used to gently grind the tissue through the mesh in the presence of cold RPMI 1640 medium supplemented with 2% FBS. The resulting cell suspension was centrifuged at 50 × g for 5 minutes at 4 °C to pellet hepatocytes. The supernatant containing non-parenchymal cells was collected and centrifuged at 300 × g for 8 minutes. The pellet was resuspended in 40% Percoll (Cat. No. BS909; Biosharp, Beijing, China) gently layered onto 70% Percoll to create a two-layer gradient. After centrifugation at 800 × g for 20 minutes at room temperature without brake, the interphase was collected, washed with PBS, and filtered again through a 40-μm cell strainer to obtain single-cell suspensions.

For flow cytometric analysis, NPCs were preincubated with anti-CD16/32 (Cat. No. 553141; BD Pharmingen, USA) to block Fcγ receptors, followed by staining with the following fluorophore-conjugated antibodies:

Alexa Fluor® 700-CD45 (BD, 560510), APC-Cy7-CD11b (BioLegend, 101225), PE-Ly6G (BioLegend, 127605), BV605-F4/80 (BioLegend, 123133), BV421-CD3ε (BD, 562600), PerCP-Cy5.5-CD19 (BioLegend, 115529), FITC-Siglec-F (BioLegend, 155504). Dead cells were excluded using 7-AAD (BD, 559925). Data were acquired on a CytoFLEX (Beckman Coulter, USA) and analyzed using FlowJo.

### Neutrophil isolation

Mouse bone marrow-derived neutrophils were isolated and purified by density gradient centrifugation as previously reported. According to the Mouse Bone Marrow Neutrophil Isolation Kit (Cat. No. P8550; Solarbio; Beijing, China) instruction, mature neutrophils are collected for further experiments.

### NET extraction and quantification

Mouse bone marrow-derived neutrophils were treated with 1 μM PMA for 6 h (PMA is widely used as a potent inducer of NET formation *in vitro* 20). After the cell culture medium was gently aspirated away, NETs adhering to the bottom were washed with 3 mL cold PBS and centrifuged at 1000 ×*g* for 10 min at 4 °C. The cell-free supernatant containing NETs was gently pipetted and stored at -20 °C after concentration determination using dsDNA HS assay Kit (Cat. No.12640ES76; Yeasen, Shanghai, China). The concentration of extracted NETs applied to AML-12 cells was 500 ng/mL.

### Neutrophil migration assay

Neutrophil migration was measured using the Boyden chamber inserts (6.5 mm, 5 μm; Ref. #3421; Corning, USA). Bone marrow-derived neutrophils cells were seeded into the upper chamber. Mouse IL-1 alpha protein, (20 ng/ml, Cat. No. HY-P7072; MCE, USA) was directly added to the conditioned media. 4 h after seeding, the inserts were lifted using forceps and washed with PBS. The cells on the inside of the inserts were gently removed using moistened cotton swabs and the cells on the lower surface of the membrane were then stained with crystal violet. The inserts were then rinsed with PBS to remove unbound dye and air-dried. The migrated cells were observed and imaged under a microscope.

### Scanning electron microscopy of net formation

Bone marrow-derived neutrophils were seeded onto 14-mm round coverslips placed in a 24-well culture plate. After stimulation with mouse IL-1α protein (20 ng/mL, Cat. No. HY-P7072; MCE, USA) for 4 hours, the cells were fixed with 5% glutaraldehyde at 4 °C for 12 hours. Following fixation, samples were washed three times with PBS and then dehydrated through a graded ethanol series (30%, 50%, 70%, 80%, 90%, and 100%, each for 20 minutes at 4 °C). Subsequently, the samples were immersed in hexamethyldisilazane for 15 minutes each. After air-drying completely in a fume hood, the samples were sputter-coated with gold to ensure conductivity. Imaging was performed using a scanning electron microscope (Gemini 300; Carl Zeiss, Oberkochen, Germany).

### Transmission electron microscopy (TEM)

Liver tissues from ASH mice were harvested and immediately fixed in 2% glutaraldehyde in 0.1 M cacodylate buffer (pH 7.4) at 4 °C overnight. Samples were then rinsed thoroughly with the same buffer and post-fixed in 1% osmium tetroxide (OsO₄) prepared in 0.1 M cacodylate buffer for 1 h at room temperature. After fixation, the tissues were dehydrated through a graded ethanol series (50%, 70%, 80%, 90%, 95%, and 100%), each step for 10 min at 25 °C. Specimens were subsequently embedded in Eponate 12 resin and polymerized at 60 °C for 48 h. Ultrathin sections (70-90 nm) were prepared using an ultramicrotome, placed on copper grids, and contrasted with 2% uranyl acetate for 15 min followed by 1% lead citrate for 10 min. Finally, sections were examined and imaged using a transmission electron microscope (HT7700, Hitachi High Technologies, Japan) operated at 80 kV.

### Cell culture and transfection

The AML-12 cells were preserved in our laboratory. AML-12 cells were cultured in DMEM/F12 (Cat. No. C11330500BT; Gibco, USA) supplemented with 1% penicillin and streptomycin (Cat. No. 15140122; Gibco, USA) and 10% FBS (Cat. No. BC-SE-FBS07; Biochannel, Nanjing, China) at 95% air and 5% CO2 incubator.

AML-12 cells were cultured to 40%-50% confluency in serum-free medium. SiRNA-AIM2 and Lipo3000 were mixed and then were transfected into cells according to the manufacturer's instructions. After 6 h, the medium was replaced with DMEM/F12 medium supplemented with 10% FBS. After siRNA transfection, cells were cultured for 24 hours to achieve AIM2 knockdown, followed by subsequent functional assays.

### Lactate dehydrogenaseassay (LDH) assay kits

The concentrations of LDH in the cell supernatant were determined using respective assay kits, according to the manufacturer's instructions.

### RNA-seq sequencing

Each group comprised three independent biological replicates. Total RNA was extracted from neutrophils derived from control and IL-1α-treated groups using TRIzol reagent (Takara Bio, Japan). RNA quantity and quality were assessed using a NanoDrop spectrophotometer and agarose gel electrophoresis to ensure high integrity and the absence of genomic DNA contamination. Qualified RNA samples were sent to Sangon Biotech Co., Ltd. (Shanghai, China) for RNA-sequencing. Sequencing libraries were prepared using the TruSeq Stranded mRNA Library Prep Kit (Illumina) after mRNA enrichment with oligo(dT) beads, and sequenced on an Illumina NovaSeq 6000 platform with 150 bp paired-end reads. Low-quality reads and adaptors were removed, and the clean reads were aligned to the mouse reference genome using HISAT2. Gene expression levels were calculated as fragments per kilobase of transcript per million mapped reads (FPKM). Differentially expressed genes (DEGs) between groups were identified using DESeq2 with thresholds of |log₂FC| ≥ 1 and adjusted P value < 0.05. A volcano plot was generated to visualize DEGs, and Kyoto Encyclopedia of Genes and Genomes (KEGG) pathway enrichment analysis was performed using the clusterProfiler R package.

### Protein extraction and western blot

Protein extraction was performed by lysing mouse liver tissue samples (30 mg), AML-12 hepatocytes, and neutrophils using RIPA lysis buffer. Equal amounts of total protein were resolved on 8-12% SDS-PAGE gels, followed by electrophoretic transfer onto PVDF membranes (Millipore, Billerica, MA, USA). Membranes were blocked at room temperature for 5 minutes using Ready-to-Use Blocking Buffer (Cat. No. YWB0501; Yoche, Shanghai, China). After blocking, membranes were incubated overnight at 4 °C with primary antibodies specific to cleaved caspase-3 (AF7022; Affinity, Beijing, China), CitH3 (Cat. No. EPR20358-120; Abcam, USA), β-actin (GB11001-100; Servicebio, Wuhan, China), and AIM2 (Cat. No. 63660; Cell Signaling Technology, USA). Following washing with TBS-T containing 0.1% Tween-20 (TBS-T), membranes were incubated with HRP-conjugated secondary antibody (Goat Anti-Rabbit/Mouse IgG-HRP, M21003; Abmart, Shanghai, China). After additional washes in TBS-T, immunoreactive bands were detected using an enhanced chemiluminescence (ECL) kit (Epizyme, Shanghai, China). Band intensity was quantified using standard densitometric analysis methods.

### RNA isolation and real-time PCR

Total RNA was isolated from mouse kidney tissues using TRIzol reagent (Invitrogen, USA), following the manufacturer's protocol. Complementary DNA (cDNA) was synthesized from purified RNA using the ThermoScript RT-PCR Synthesis Kit (AG, Hunan, China). Quantitative real-time PCR (qRT-PCR) was subsequently conducted with the ThermoScript RT-qPCR kit on an ABI StepOnePlus Real-Time PCR System (Applied Biosystems, Foster City, CA, USA). PCR amplification was carried out using SYBR Green-based detection chemistry with gene-specific primers. Relative mRNA expression levels were determined using the 2^-ΔΔCt^ method, with primer sequences listed in with primer sequences listed in Table S1.

### Statistical analysis

All data are presented as the mean ± standard error of the mean (SEM), based on at least three independent experiments. Statistical significance between two groups was assessed using a two-tailed unpaired Student's *t*-test. Statistical significance between multiple group comparisons, one-way or two-way analysis of variance (ANOVA) was performed, followed by Tukey's post hoc test where appropriate. Categorical variables were expressed as counts. All statistical analyses and graphical presentations were conducted using GraphPad Prism software (version 8.0; San Diego, CA, USA). A p-value < 0.05 was considered statistically significant, and all tests were two-sided.

## Results

### NETs formation aggravates liver injury in ASH

**Figure 1:**
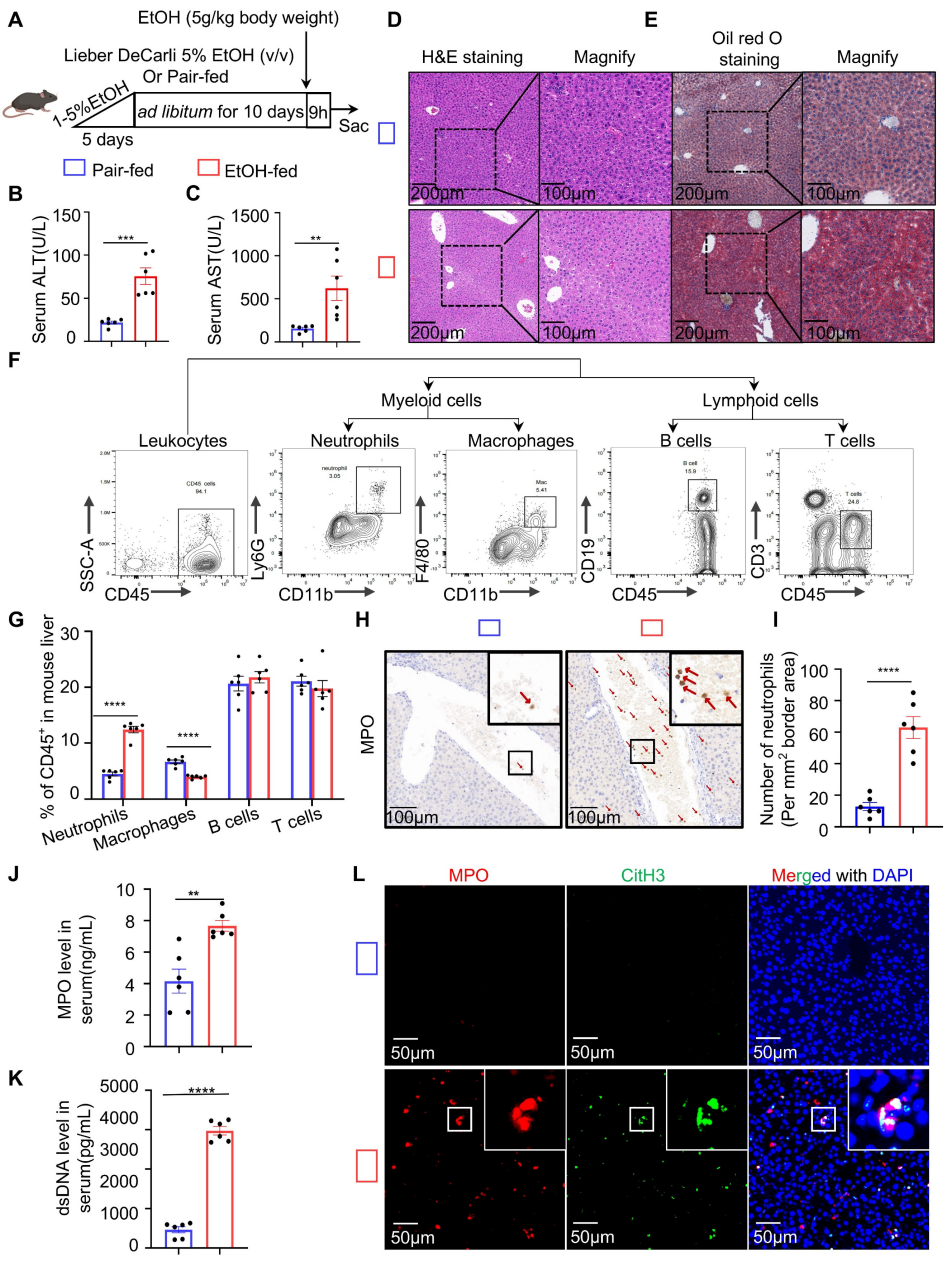
** Chronic-binge ethanol feeding induces liver injury, steatosis, immune cell infiltration, and NETs formation in ASH mice.** (A) Schematic illustration of the chronic-binge ethanol feeding protocol used to induce alcohol-associated steatohepatitis (ASH) in mice. (B, C) Serum alanine aminotransferase (ALT) and aspartate aminotransferase (AST) levels were significantly elevated in ethanol (EtOH)-fed mice (n = 6 per group). (D, E) Representative liver sections stained with hematoxylin and eosin (H&E) (D) and Oil Red O (E) showing hepatocellular ballooning, architectural distortion, and lipid accumulation in EtOH-fed mice. Original images, scale bar = 200 μm; magnified insets, scale bar = 100 μm. (F) Gating strategy for flow cytometric analysis of hepatic immune cell subsets, including neutrophils (CD11b⁺Ly6G⁺), macrophages (CD11b⁺F4/80⁺), B cells (CD45⁺CD19⁺), and T cells (CD45⁺CD3⁺). (G) Quantification of CD45⁺ hepatic immune cell subsets, showing significantly increased neutrophils and macrophages in EtOH-fed mice (n = 6 per group). (H, I) Immunohistochemical staining for Myeloperoxidase (MPO) showing increased neutrophil infiltration in EtOH-fed livers. Red arrows indicate MPO⁺ neutrophils. Scale bar = 100 μm. Neutrophils were quantified in five randomly selected fields per sample (n = 6 per group). (J, K) Serum levels of MPO and double-stranded DNA (dsDNA) were significantly elevated in EtOH-fed mice, indicative of NETs formation (n = 6 per group). (L) Multiplex immunofluorescence staining of liver tissues for MPO (red), citrullinated histone H3 (CitH3; green), and DNA (DAPI, blue) highlighting extracellular NET structures in EtOH-fed mice. Scale bar = 50 μm. Data are presented as mean ± SEM. **P < 0.01, ***P < 0.001, ****P < 0.0001 by two-tailed unpaired Student's *t*-test.

To explore the immune response in ASH disease, we utilized the chronic-binge ethanol feeding model, which faithfully mimics the drinking behavior of human alcoholics and reproduces pathological features characteristic of early-stage ASH 21 (Figure 1A). Serological analysis revealed that ethanol (EtOH)-fed mice exhibited significantly elevated levels of liver enzymes, including ALT and AST (Figure 1B and 1C). Histological examination by H&E staining showed marked hepatocellular ballooning, disorganized hepatic architecture, and extensive lipid vacuolization in the livers of EtOH-fed mice (Figure 1D). Oil Red O staining confirmed the accumulation of abundant intracellular lipid droplets in EtOH group, as evidenced by prominent red staining in hepatocytes (Figure 1E). These findings indicate that the ASH model mice developed hepatic dysfunction and lipid metabolism disorders. To assess hepatic immune cell infiltration, we analyzed the proportions of major immune cell populations in the livers of ASH mice. Flow cytometry revealed a significant increase in the proportion of neutrophils in the EtOH-fed group relative to controls (Figure 1F and 1G). IHC staining using MPO, a neutrophil marker, demonstrated extensive neutrophil infiltration around the portal veins and within the hepatic lobules of EtOH-fed mice (Figure 1H). Quantification of IHC staining confirmed a significantly increased number of neutrophils per unit area in the model group (Figure 1I). To determine whether the infiltrating neutrophils formed NETs, we measured serum levels of neutrophil-derived components, including MPO and cell-free dsDNA, both indicative of NET formation (Figure 1J and 1K). CitH3, a key component of NETs involved in chromatin decondensation and extracellular DNA scaffold formation, was used as a specific marker. Multiplex immunofluorescence staining with DAPI (used for DNA staining), MPO, and CitH3 revealed a substantial presence of NETs in the liver tissues of ASH mice (Figure 1L).

**Figure 2:**
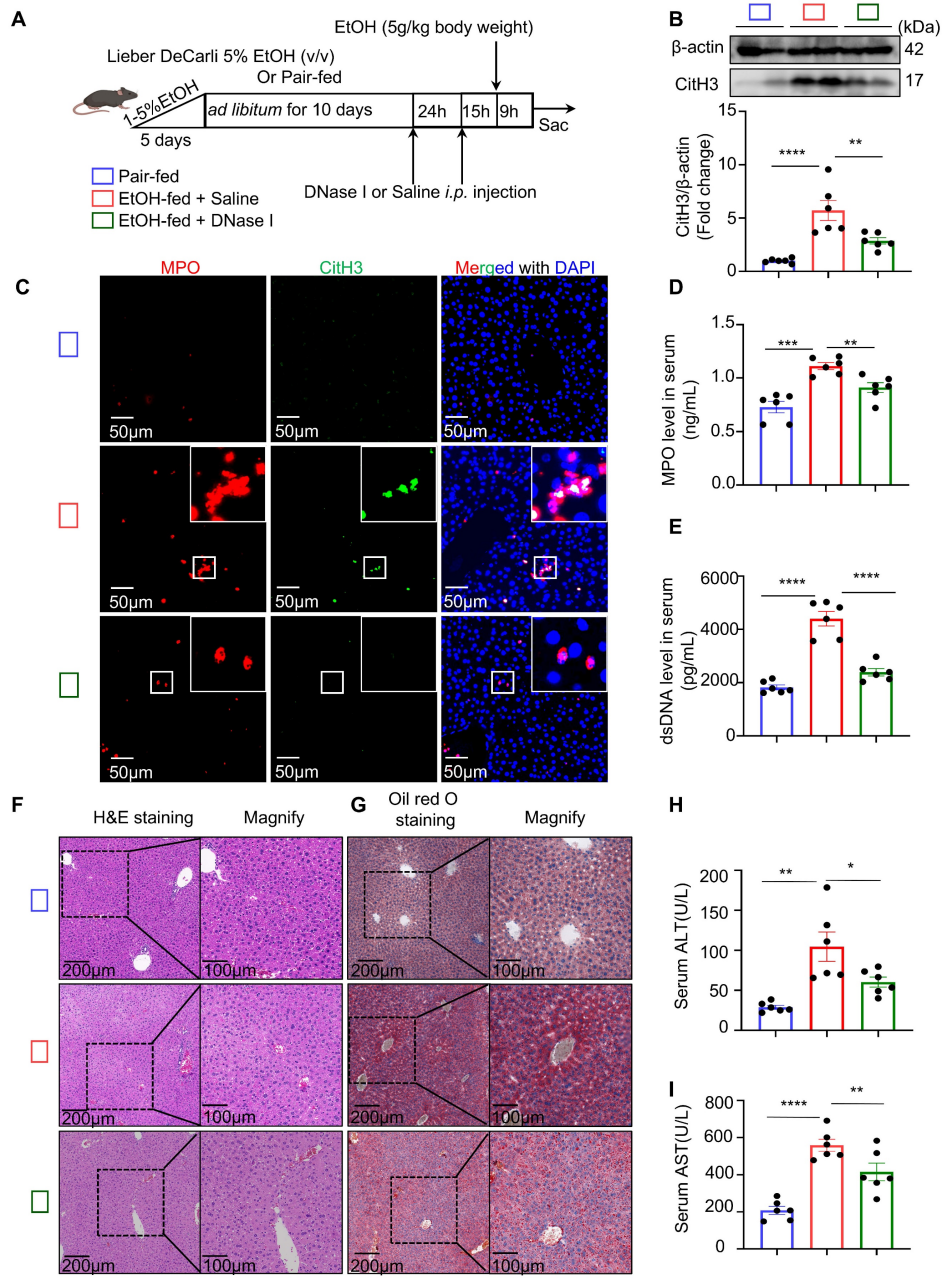
** DNase I treatment degrades NETs and alleviates liver injury in ASH mice.** (A) Schematic illustration of the experimental design. Mice were fed a Lieber-DeCarli EtOH diet or pair-fed control diet for 10 days, followed by DNase I (10 U in 100 μL 0.9% NaCl, intraperitoneally [i.p.]) or Saline intraperitoneal injection at 24 and 15 hours prior to tissue harvest. DNase I or vehicle was administered to assess the impact of NET degradation. (B) Western blot analysis of CitH3 in liver tissues. Representative blots and densitometric quantification are shown (n = 6 per group). (C) Representative multiplex immunofluorescence images of liver sections stained for MPO (red), CitH3 (green), and DAPI (blue) in liver sections. Insets highlight colocalized NET structures. Scale bar = 50 μm. (D, E) Quantification of serum MPO (D) and dsDNA (E) levels in different treatment groups (n = 6 per group). (F, G) Representative images of H&E (F) and Oil Red O staining (G) of liver sections, showing histological injury and lipid accumulation, respectively. Original images, scale bar = 200 μm; magnified insets, scale bar = 100 μm. (H, I) Serum ALT (H) and AST (I) levels were significantly reduced in DNase I-treated mice (n = 6 per group). Data in figures (B, D, E, H and I) are presented as mean ± SEM. *P < 0.05, **P < 0.01, ***P < 0.001, ****P < 0.0001 by one-way ANOVA with Tukey's multiple comparisons test.

To investigate whether NETs are a major contributor to neutrophil-mediated liver injury in ASH mice, DNase I—a deoxyribonuclease capable of degrading extracellular DNA—was administered to degrade NETs *in vivo* (Figure 2A). Following DNase I treatment, the hepatic levels of CitH3 were markedly reduced (Figure 2B). Multiplex immunofluorescence staining further confirmed that NETs structures were nearly absent in the livers of DNase I-treated mice (Figure 2C). In addition, serum analysis showed that DNase I significantly decreased the concentrations of both MPO and dsDNA (Figure 2D and 2E). These results collectively indicate that NETs were successfully cleared in ASH mice following DNase I treatment. We next assessed whether DNase I treatment ameliorated lipid metabolic dysregulation and hepatic injury. H&E and Oil Red O staining (Figure 2F and 2G) demonstrated an improvement in hepatic lipid accumulation in DNase I-treated mice. Moreover, serum ALT and AST (Figure 2H and 2I) levels were significantly decreased, suggesting a partial restoration of liver function. These results suggest that NETs contribute to ASH-associated liver injury and steatosis.

### NETs promote hepatocyte apoptosis under ethanol-induced sublethal injury

**Figure 3:**
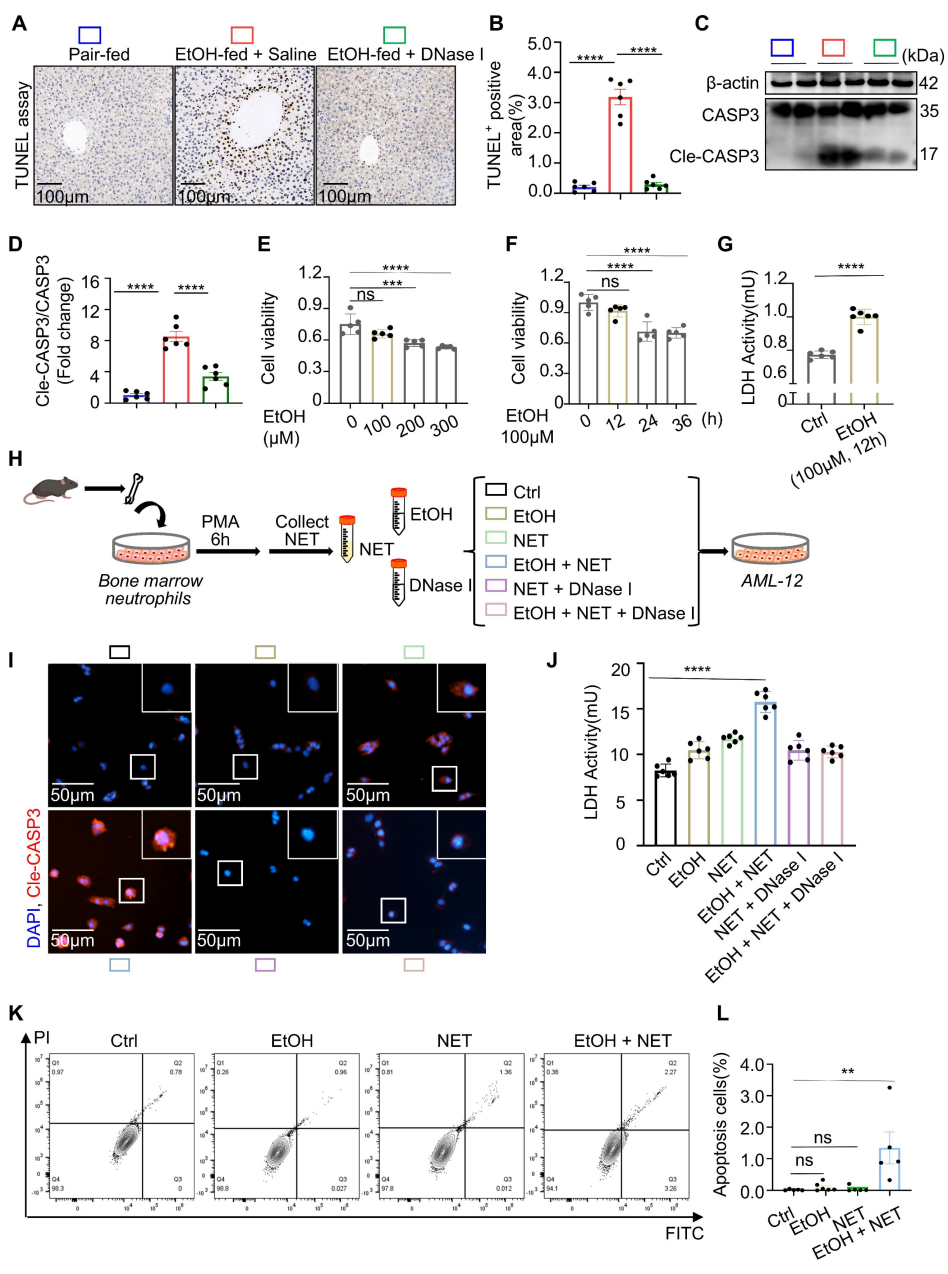
** NETs promote hepatocyte apoptosis under ethanol-induced sublethal injury.** (A) Representative TUNEL staining images of liver sections from pair-fed, EtOH-fed, and EtOH-fed + DNase I-treated mice. Scale bar = 50 μm. (B) Quantification of TUNEL-positive hepatocyte area showed that DNase I significantly reduced ethanol-induced apoptosis (n = 6 per group). (C, D) Western blot and densitometric analysis of cleaved caspase-3 (Cle-CASP3) in liver lysates. DNase I markedly reduced caspase-3 activation (n = 6/group). (E, F) CCK-8 assays revealed that ethanol up to 100 µM and within 12 h exposure did not induce apoptosis in AML-12 hepatocytes (n = 6 per group). (G) LDH assays showed that 100 µM ethanol for 12 h caused sublethal AML-12 hepatocyte injury (n = 6 per group). (H) Experimental workflow illustrating NETs collection from PMA-stimulated bone marrow neutrophils and their co-incubation with AML-12 cells under different treatment conditions. (I) Representative immunofluorescence staining of cleaved caspase-3 (red) and nuclei (DAPI, blue) in AML-12 cells treated with EtOH, NETs, or both. Scale bar = 50 μm. (J) LDH release assays demonstrated that NETs, especially when combined with ethanol, significantly aggravated hepatocyte damage (n = 6 per group). (K, L) Flow cytometry with FITC/PI staining revealed increased late apoptotic cells upon combined EtOH + NETs exposure. Statistical analysis confirmed enhanced apoptosis compared with other groups (n = 6 per group). Data are presented as mean ± SEM. **P < 0.01, ***P < 0.001, ****P < 0.0001; ns, not significant; Statistical significance was determined by one-way ANOVA with Tukey's multiple comparisons test (B, D, E, F, J, L), or two-tailed unpaired Student's *t*-test (G), as appropriate.

Hepatocytes account for approximately 80% of all liver cells and are the primary executors of hepatic function 22. Extensive hepatocyte death leads to impaired liver function. In the following experiments, we investigated the effect of neutrophils on hepatocyte death. TUNEL staining, which labels fragmented DNA, was used to detect apoptotic cells. Liver tissues from control, ASH model, and DNase I-treated mice were subjected to TUNEL staining. TUNEL-positive cells typically exhibited nuclear condensation without significant disruption of the cell membrane (Figure 3A). Based on these morphological features characteristic of programmed cell death, we concluded that hepatocyte death in ASH mice occurred primarily via apoptosis. Semi-quantitative analysis of TUNEL staining showed that DNase I treatment significantly reduced hepatocyte apoptosis in ASH mice (Figure 3B). Cleaved caspase-3, a classical and early marker of apoptosis, was markedly upregulated in ASH model mice and significantly decreased after DNase I administration (Figure 3C and 3D). These findings further suggest that NETs may contribute to apoptosis in ASH.

In ASH mice, hepatocytes undergoing apoptosis are often subjected to dual stress from both ethanol and NETs. For these cells, ethanol alone serves as a non-lethal stimulus. To better simulate the pathological context of this subpopulation of hepatocytes *in vivo*, we utilized AML-12 cells 23—a well-established hepatocyte cell line widely used in ALD research—to identify ethanol conditions that cause hepatocellular injury without inducing apoptosis. CCK-8, which reflects cell viability via intracellular dehydrogenase activity, and LDH, which is released by damaged cells into the culture supernatant, were jointly used to evaluate non-apoptotic injury conditions. We first used CCK-8 assays to assess the apoptotic potential of various ethanol concentrations over a 12-hour period and determined that concentrations up to 100 μM were non-apoptotic (Figure 3E). Using 100 μM ethanol, we then tested different durations of stimulation and confirmed that exposure up to 12 hours did not induce apoptosis (Figure 3F). LDH assays revealed that ethanol at 100 μM for 12 hours caused significant cellular damage (Figure 3G). This condition was subsequently used as the standard sub-apoptotic ethanol injury model in our following experiments.

To explore the direct effect of NETs on hepatocytes, we next examined their impact under the established ethanol injury conditions. Neutrophils were isolated from mouse bone marrow and stimulated with PMA to induce and purify NET-derived DNA (Figure 3H). Immunofluorescence staining of cleaved caspase-3 revealed that NETs alone induced hepatocyte apoptosis, and this effect was further exacerbated when combined with ethanol (Figure 3I). LDH assays of the culture supernatant confirmed that NETs together with ethanol caused severe hepatocellular damage (Figure 3J). Flow cytometric analysis using propidium iodide (PI) staining showed that the majority of affected cells were in the late apoptotic stage under the combined treatment (Figure 3K and 3L). Through systematic evaluation of NETs under different conditions in ethanol-injured hepatocytes, we confirmed that NETs are a key factor mediating hepatocyte apoptosis.

### Ethanol-injured hepatocytes release IL-1α to trigger neutrophil recruitment and NETs release

**Figure 4:**
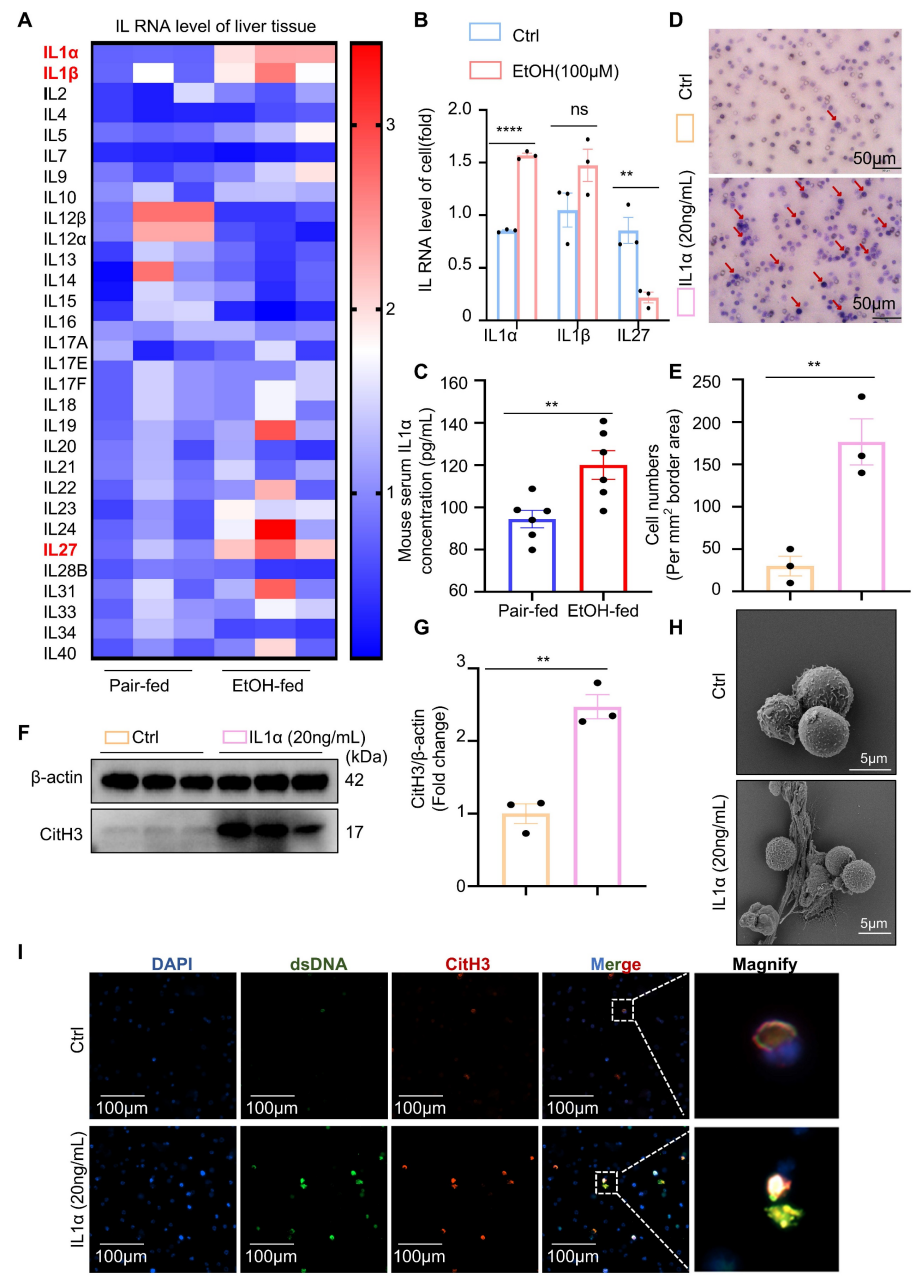
** Hepatocyte-derived IL-1α mediates neutrophil chemotaxis and NETs formation.** (A) Heatmap showing hepatic mRNA expression of 30 interleukins in pair-fed and EtOH-fed mice, with IL-1α, IL-1β, and IL-27 notably upregulated in the EtOH-fed group (n = 3 per group). (B) qRT-PCR analysis of IL-1α, IL-1β, and IL-27 mRNA expression in AML-12 cells treated with or without 100 μM EtOH for 12 h (n = 3 per group). (C) Serum IL-1α concentrations were significantly elevated in EtOH-fed mice compared to pair-fed controls (n = 6 per group). (D, E) Transwell migration assay showing neutrophil chemotaxis toward 20 ng/mL IL-1α. Migrated cells were stained with crystal violet and quantified as the number of cells per mm² (n = 3 per group). Scale bar = 100 μm. (F, G) Western blot and densitometric quantification of CitH3 in neutrophils treated with or without 20 ng/mL IL-1α for 4 h (n = 3 per group). (H) Scanning electron microscopy reveals extracellular web-like structures indicative of NETs in neutrophils exposed to IL-1α. Scale bar = 5 μm. (I) Immunofluorescence staining of neutrophils with DAPI (blue), anti-dsDNA (green), and anti-CitH3 (red) antibodies confirms NETs release following IL-1α stimulation. Scale bar = 100 μm. Data are presented as mean ± SEM. **P < 0.01, ****P < 0.0001; ns, not significant. Statistical significance was assessed by two-tailed unpaired Student's *t*-test (B, C, E, G).

In the preceding experiments, we demonstrated that neutrophils induce apoptosis in hepatocytes under ethanol stress via the formation of NETs. However, the DAMPs released by injured hepatocytes that trigger neutrophil responses remain unclear. ILs are a family of key signaling molecules that regulate immune cell chemotaxis and activation. To identify interleukins potentially involved in ASH-related immune regulation, we assessed the hepatic mRNA expression of 30 interleukins in pair-fed and EtOH-fed mice (Figure 4A). The results revealed significant upregulation of IL-1α, IL-1β, and IL-27 in the livers of EtOH-fed mice, suggesting their potential roles in modulating immune activity during ASH progression. We next examined the expression of these three interleukins in normal hepatocytes and hepatocytes injured by ethanol exposure. Among them, only IL-1α was significantly upregulated in damaged hepatocytes (Figure 4B), indicating that IL-1α may serve as a DAMP produced by entanol-injured hepatocytes. Consistently, IL-1α levels were also elevated in the serum of EtOH-fed mice compared to pair-fed controls (Figure 4C), further supporting its role as a hepatocyte-derived DAMP conveying injury signals to immune cells. To assess the effect of IL-1α on neutrophil behavior, we performed Transwell migration assays. Neutrophils exhibited pronounced chemotaxis toward the lower chamber containing 20 ng/mL IL-1α (Figure 4D and 4E), confirming that IL-1α promotes neutrophil migration. Additionally, IL-1α stimulation induced robust citrullination of histones in neutrophils (Figure 4F and 4G). Scanning electron microscopy revealed the presence of extracellular web-like structures in neutrophils treated with IL-1α (Figure 4H). To confirm the identity of these structures as NETs, we used anti-dsDNA and anti-CitH3 antibodies to co-label extracellular citrullinated double-stranded DNA. Immunofluorescence staining clearly demonstrated that IL-1α induces the release of NETs from neutrophils (Figure 4I). Collectively, these results identify IL-1α as a critical DAMP released by ethanol-injured hepatocytes, which promotes neutrophil migration and activation, ultimately triggering NET formation.

### Hepatic IL-1α modulates NET formation and exacerbates liver injury in ASH

**Figure 5:**
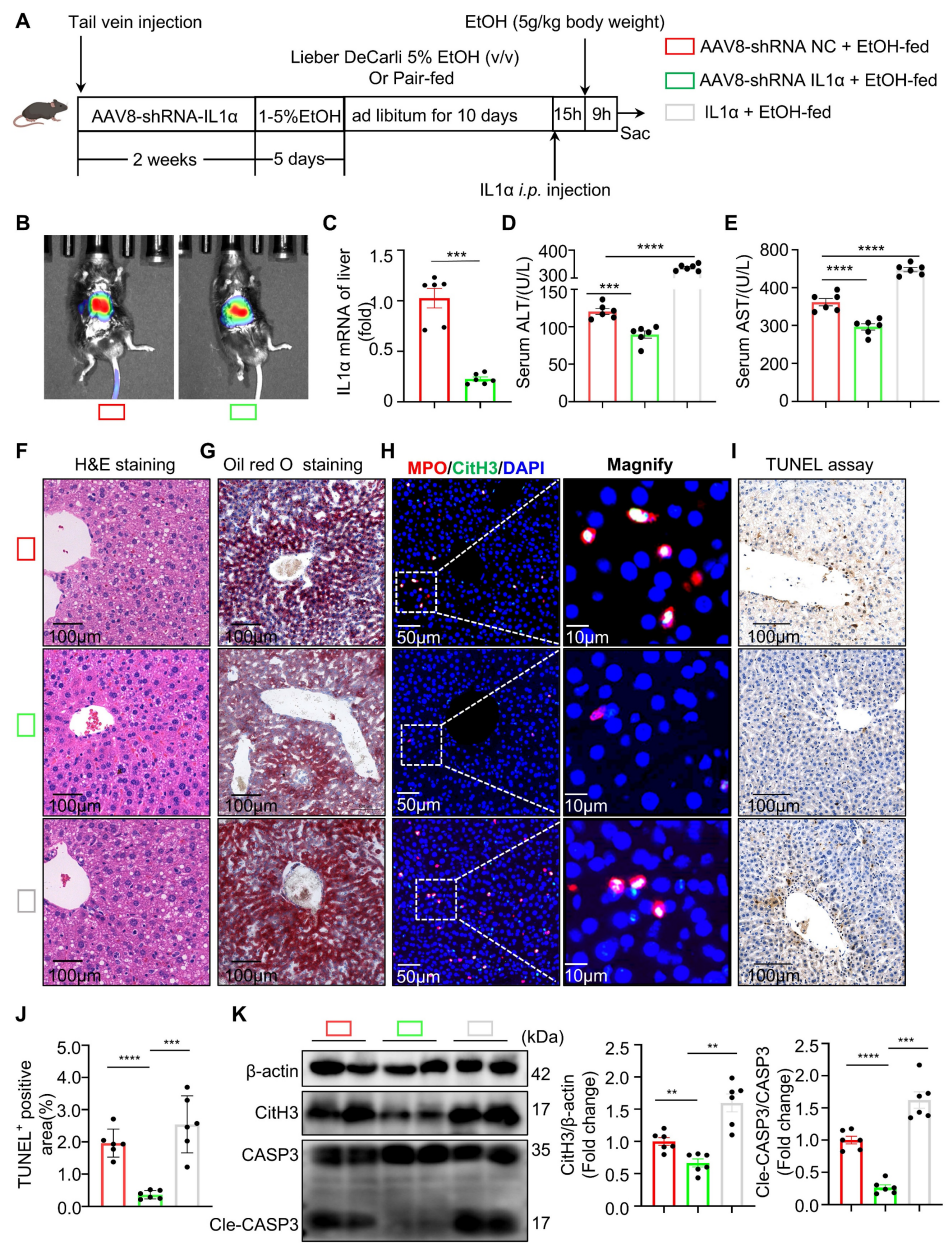
** Hepatic IL-1α modulates NET formation and exacerbates liver injury in ASH.** (A) Schematic of experimental design: mice received either AAV8-shRNA targeting IL-1α or control vector via tail vein injection, followed by Lieber-DeCarli ethanol feeding; recombinant IL-1α (10 μg/kg, diluted in 0.9% NaCl) was administered via intrahepatic injection 15 h prior to euthanasia. (B) Representative *in vivo* bioluminescent imaging showing liver-specific expression of AAV8-shRNA vectors. (C) Quantification of IL-1α mRNA expression in liver tissue confirmed effective knockdown in AAV8-shRNA-IL-1α-treated mice (n = 6 per group). (D, E) Serum ALT and AST levels were significantly decreased in IL-1α knockdown mice and increased in IL-1α-injected mice compared to AAV8-shRNA negative controls (n = 6 per group). (F, G) H&E and Oil Red O staining of liver sections revealed reduced steatosis in IL-1α-silenced mice and increased lipid accumulation in the IL-1α-injected group. Scale bar = 100 μm. (H) Immunofluorescence staining for MPO (red), CitH3 (green), and DAPI (blue) in liver sections indicated that NET formation correlated with hepatic IL-1α expression. Original images, scale bar = 50 μm; magnified insets, scale bar = 10 μm. (I, J) Representative TUNEL staining and quantification of apoptotic area, revealing IL-1α-dependent enhancement of hepatocyte apoptosis (n = 6 per group). Scale bar = 100 μm. (K) Western blot analysis and quantification of CitH3 and cleaved caspase-3 (Cle-CASP3) levels in liver lysates, showing increased NETs and apoptosis with elevated IL-1α (n = 6 per group). Data are presented as mean ± SEM. *P < 0.05, **P < 0.01, ***P < 0.001, ****P < 0.0001; ns, not significant. Statistical significance was determined by one-way ANOVA with Tukey's multiple comparisons test (D, E, J, K), or two-tailed unpaired Student's* t*-test (C), as appropriate.

Whether suppression of hepatocyte-derived IL-1α could reduce intrahepatic NET formation and alleviate ASH progression requires further investigation. To explore this hypothesis, we utilized an adeno-associated virus serotype 8 (AAV8)-packaged IL-1α shRNA plasmid delivered via tail vein injection to silence hepatic IL-1α expression in mice. Conversely, recombinant mouse IL-1α protein was directly administered into the liver through in situ injection to enhance hepatic IL-1α levels (Figure 5A). *In vivo* imaging confirmed successful hepatic expression of AAV8-shRNA IL-1α four weeks post tail vein injection (Figure 5B). Subsequent assays confirmed effective knockdown of IL-1α expression in liver tissues of the AAV8-shRNA IL-1α-treated group (Figure 5C). Compared with the control AAV8-shRNA NC+EtOH-fed group, mice in the IL-1α knockdown group exhibited significantly lower serum ALT and AST levels, whereas IL-1α-injected mice showed a marked increase of these two liver function parameters (Figure 5D and 5E), indicating severe hepatic damage associated with elevated hepatic IL-1α levels. H&E and Oil Red O staining revealed that hepatic IL-1α elevation exacerbated liver steatosis (Figure 5F and 5G). Immunofluorescence co-staining for MPO and CitH3 demonstrated widespread NET formation in liver tissues from both AAV8-shRNA NC+EtOH-fed and IL-1α-injected EtOH-fed mice (Figure 5H), suggesting that increased hepatic IL-1α promotes intrahepatic NET generation. TUNEL staining further indicated enhanced hepatocyte apoptosis in the presence of elevated IL-1α (Figure 5I and 5J). Consistently, hepatic expression of CitH3 and cleaved caspase-3 proteins also correlated positively with intrahepatic IL-1α levels (Figure 5K), reinforcing the association between hepatic IL-1α, NET formation, and hepatocyte apoptosis. Collectively, our findings support the hypothesis that suppression of hepatic IL-1α production could reduce intrahepatic NET formation, thereby ameliorating liver injury in ASH mice.

### Neutrophil TLR9 acts downstream of IL-1α to promote NET formation in ASH

**Figure 6:**
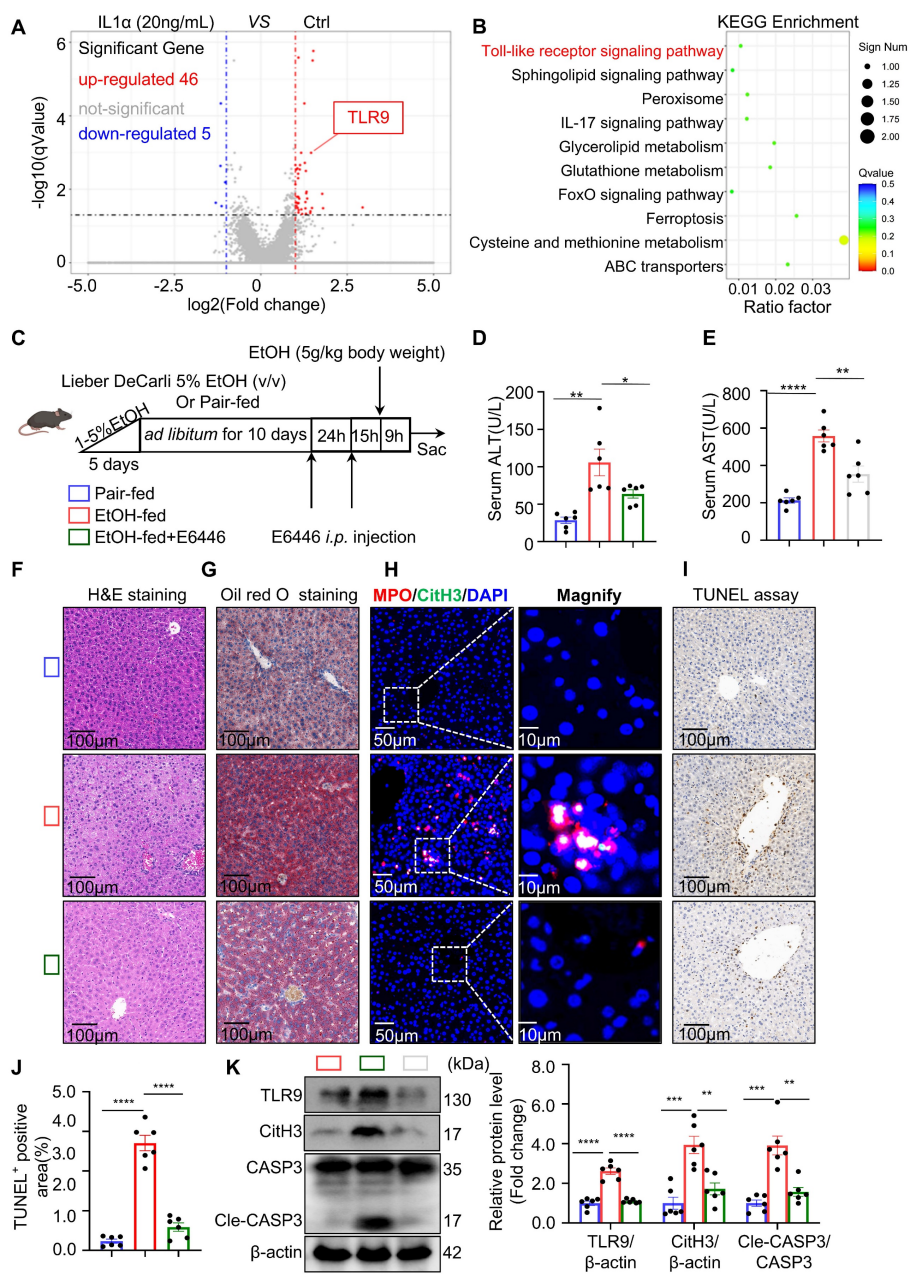
** TLR9 acts downstream of IL-1α to mediate NET formation and hepatocyte apoptosis in ASH.** (A) Volcano plot of RNA-seq data showing differentially expressed genes in neutrophils treated with IL-1α compared to untreated controls. TLR9 was among the significantly upregulated genes (red dots, n = 3 per group). (B) KEGG pathway enrichment analysis of 46 upregulated genes identified Toll-like receptor signaling as the most enriched pathway. (C) Experimental timeline. Mice were pair-fed or fed an ethanol-containing Lieber-DeCarli diet for 10 days. E6446 (a TLR9 inhibitor, 20 mg/kg, orally [p.o.]) was administered 24 and 15 hours prior to euthanasia. (D, E) Serum ALT and AST levels were significantly reduced in EtOH-fed mice treated with E6446 compared to untreated ASH mice (n = 6 per group). (F, G) Representative H&E and Oil Red O staining of liver sections showing improved histology and reduced lipid accumulation in the E6446 group. Scale bar = 100 μm. (H) Immunofluorescence staining of liver tissues showing reduced intrahepatic NET formation (MPO: red; CitH3: green; DAPI: blue) after E6446 treatment. Original images, scale bar = 50 μm; magnified insets, scale bar = 10 μm. (I, J) Representative TUNEL staining and quantification of TUNEL-positive areas showed a reduction in hepatocyte apoptosis in E6446-treated mice (n = 6 per group). Scale bar = 100 μm. (K) Western blot and densitometric quantification of hepatic TLR9, CitH3, and Cle-CASP3 levels. E6446 treatment significantly reduced expression of all three proteins (n = 6 per group). Data are presented as mean ± SEM. *P < 0.05, **P < 0.01, ***P < 0.001, ****P < 0.0001; ns, not significant. Statistical comparisons were performed using one-way ANOVA followed by Tukey's multiple comparisons test.

Next, we investigated the molecular mechanism by which hepatocyte-derived IL-1α promotes NET formation in neutrophils. RNA sequencing was performed to comprehensively profile transcriptomic changes in neutrophils following IL-1α stimulation. Volcano plot analysis of the RNA-seq data revealed that TLR9—a well-characterized DNA-sensing receptor previously implicated in NET formation—was significantly upregulated in IL-1α-treated neutrophils (Figure 6A). To further characterize the biological functions of IL-1α-responsive genes, KEGG pathway enrichment analysis was conducted on the 46 upregulated genes. Notably, pathways associated with NET formation were significantly enriched in the IL-1α group (Figure 6B), suggesting a transcriptional activation of NET-related programs.

Our previous study demonstrated that pharmacological inhibition of TLR9 *in vitro* effectively suppresses NETs formation 13. However, the impact of TLR9 inhibition on ASH pathogenesis *in vivo* remains unclear. To address this, we administered E6446, a selective TLR9 inhibitor, via intraperitoneal injection to EtOH-fed mice (Figure 6C). Mice treated with E6446 exhibited significantly reduced serum ALT and AST levels compared to untreated ASH model mice (Figure 6D and 6E), indicating an improvement in liver function. Histological analysis using H&E and Oil Red O staining (Figure 6F and 6G) revealed reduced lipid accumulation in hepatocytes following E6446 treatment. Dual immunofluorescence staining for MPO and CitH3 (Figure 6H) showed a marked reduction in intrahepatic NETs formation in E6446-treated mice. Serum levels of MPO and dsDNA were significantly decreased in the E6446-treated group relative to the ASH model group (Figure S1A and S1B), indicating reduced NET formation *in vivo*. Consistently, TUNEL staining (Figure 6I and 6J) revealed fewer apoptotic hepatocytes in the E6446 group. Western blot (Figure 6K) analysis of liver tissues confirmed that TLR9 expression was significantly decreased in E6446-treated mice. Moreover, the expression levels of CitH3, a NETs marker, and cleaved caspase-3, a key apoptosis marker, were also substantially reduced. Taken together, these findings suggest that TLR9 is a critical downstream effector of IL-1α signaling in neutrophils, mediating NETs formation during ASH progression. Pharmacological inhibition of TLR9 effectively attenuates NETs formation, hepatocyte apoptosis, and liver injury.

Under the influence of IL-1α, increased expression of the TLR9 receptor suggests that neutrophils may adopt a more responsive phenotype with enhanced potential for NET formation. Previous studies have shown that SiglecF⁺ neutrophils possess a greater propensity to form NETs compared to conventional neutrophils 24, 25. To investigate whether IL-1α can promote this phenotypic shift, we performed flow cytometry to examine the effect of IL-1α treatment on SiglecF⁺ neutrophil induction *in vitro*. The results showed that IL-1α partially converted neutrophils into the SiglecF⁺ phenotype, comparable to previously reported inducers such as TGF-β and GM-CSF 24 (Figure S2A and 2B). We further analyzed liver tissues from control and ASH model mice and found that the proportion of hepatic SiglecF⁺ neutrophils was significantly increased in the ASH group (Figure S2C and 2D). Notably, the magnitude of this increase was similar to the proportion observed in IL-1α-treated neutrophils *in vitro*. Moreover, when neutrophils were co-treated with IL-1α and the TLR9 inhibitor E6446, IL-1α no longer induced the SiglecF⁺ phenotype (Figure S2E and 2F). These findings suggest that IL-1α may regulate neutrophil phenotype through a TLR9-dependent pathway, highlighting a potential mechanism by which IL-1α modulates neutrophil activation and NET formation in ASH.

### AIM2 mediates NET-induced hepatocyte apoptosis in ASH

The cellular mechanisms by which NETs induce apoptosis in ethanol-injured hepatocytes remain unclear. Previous studies have suggested that dsDNA within NETs may activate intracellular dsDNA sensors in parenchymal cells, which in turn trigger cell death pathways. To investigate this, we first analyzed the hepatic mRNA expression levels of several dsDNA sensors—AIM2, IFI16, cGAS, and NLRP3—in control and ASH model mice. Among them, AIM2 was the most significantly upregulated sensor in the livers of ASH mice (Figure S3A). Western blot analysis further confirmed that AIM2 protein levels were markedly elevated in liver tissues of the model group (Figure S3B), suggesting a potential role for AIM2 in ASH pathogenesis.

**Figure 7:**
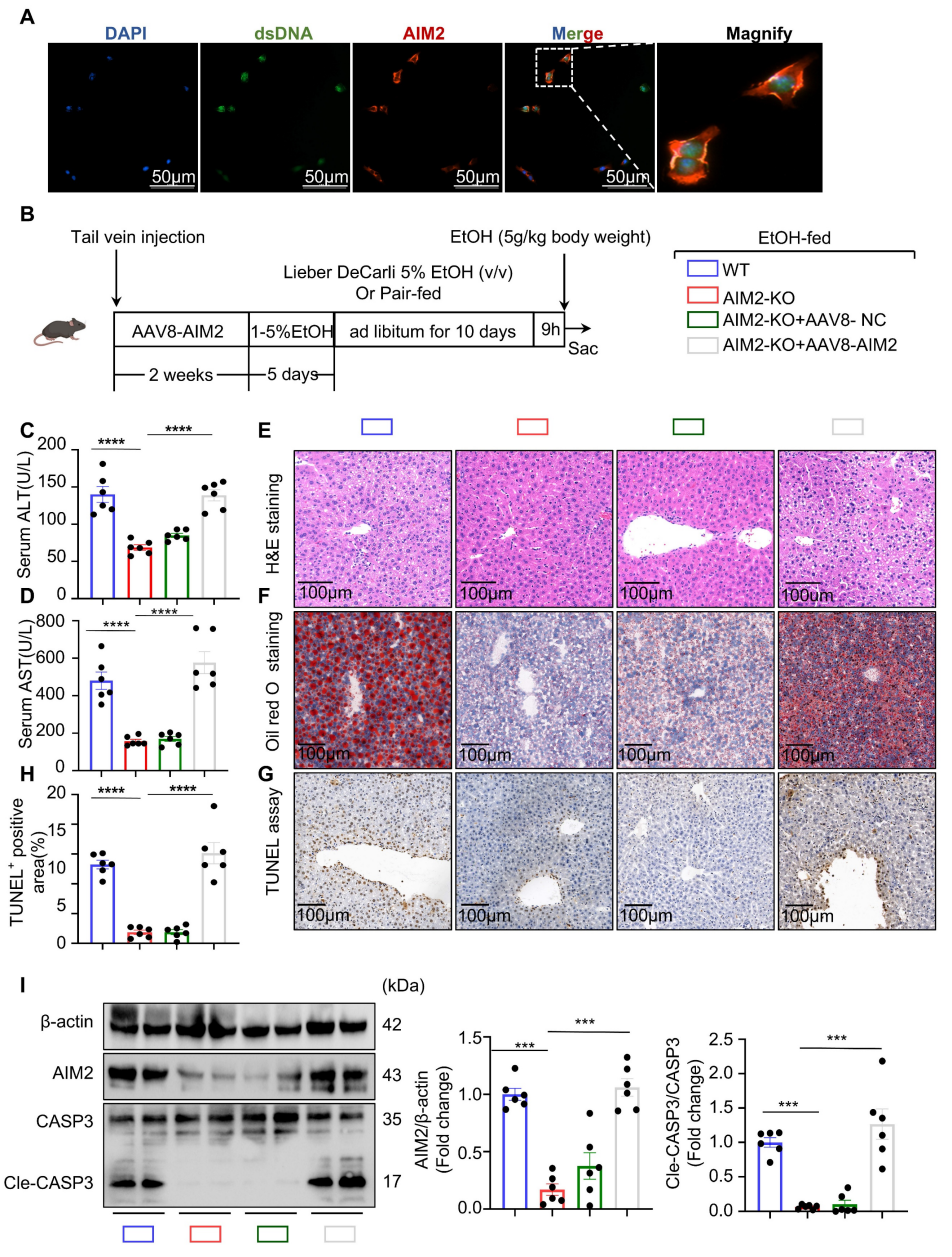
** AIM2 mediates NET-induced hepatocyte apoptosis and liver injury in ASH.** (A) Immunofluorescence staining showing co-localization of AIM2 (red) with NET-derived dsDNA (green) in ethanol-injured AML-12 hepatocytes. Nuclei were counterstained with DAPI (blue). Magnified insets highlight AIM2/dsDNA overlap. (B) Schematic of experimental design. WT or AIM2-KO mice received AAV8-NC or AAV8-AIM2 via tail vein injection, followed by Lieber-DeCarli ethanol feeding to establish the ASH model. (C, D) Serum ALT and AST levels were significantly decreased in AIM2-KO mice and restored by AAV8-mediated hepatic AIM2 reconstitution (n = 6 per group). (E, F) H&E and Oil Red O staining of liver sections showed that histological damage and lipid accumulation were associated with hepatic AIM2 expression levels. Scale bar = 100 μm. (G, H) Representative TUNEL staining and quantification of apoptotic areas in liver tissues revealed reduced hepatocyte apoptosis in AIM2-deficient mice (n = 6 per group). Scale bar = 100 μm. (I) Western blot and densitometric quantification of hepatic AIM2, CASP3, and Cle-CASP3 expression. AIM2 reconstitution enhanced cleaved CASP3 expression in the ASH model (n = 6 per group). Data are presented as mean ± SEM. *P < 0.05, **P < 0.01, ***P < 0.001, ****P < 0.0001; ns, not significant. Statistical comparisons were performed using one-way ANOVA with Tukey's multiple comparisons test.

To trace the interaction between NETs and hepatocytes, we pre-labeled isolated NETs with SYTOX Green, allowing us to track NET-derived dsDNA in subsequent experiments while avoiding signal interference from other DNA sources. The labeled NETs were added to ethanol-injured hepatocytes. After 12 hours, AIM2 immunofluorescence staining was performed on the hepatocytes. As shown in Figure 7A, AIM2 co-localized with NETs, indicating that AIM2 may recognize NETs-derived DNA in hepatocytes. To determine whether AIM2 is essential for NET-induced apoptosis, we used siRNA to knock down AIM2 in hepatocytes. Notably, the pro-apoptotic effect of NETs on ethanol-injured hepatocytes was abolished following AIM2 silencing (Figure S3C). These findings suggest that NETs activate AIM2 in hepatocytes, thereby inducing apoptosis. AIM2 likely serves as the terminal effector in the communication axis between neutrophils and injured hepatocytes in the ASH model.

Given that hepatocyte AIM2 is a critical receptor mediating NET effects, we hypothesized that AIM2 plays an important role in the progression of ASH. To evaluate this, we generated AIM2 global knockout KO mice (Figure S4A and S4B) and performed liver-specific reconstitution of AIM2 using an AAV8-packaged AIM2-expressing plasmid delivered via tail vein injection (Figure 7B). *In vivo* imaging (Figure S4C) and liver tissue analysis (Figure 7I) confirmed successful hepatic expression of AIM2 four weeks after AAV8 administration. Under Binge model feeding, serum ALT and AST levels were significantly reduced in AIM2 KO mice, while restoration of AIM2 expression in the liver led to a marked increase in these liver function markers (Figure 7C and 7D), indicating a direct association between hepatic AIM2 levels and liver injury. H&E and Oil Red O staining further showed that the degree of hepatocellular steatosis correlated with AIM2 expression levels (Figure 7E and 7F). In addition, TUNEL staining and cleaved caspase-3 analysis revealed that reduced AIM2 expression mitigated hepatocyte apoptosis in the ASH model (Figure 7G and 7I). Taken together, these findings demonstrate that suppressing hepatocyte AIM2 attenuates NETs-induced apoptosis in ethanol-injured livers and alleviates liver damage in ASH.

## Discussion

**Figure 8:**
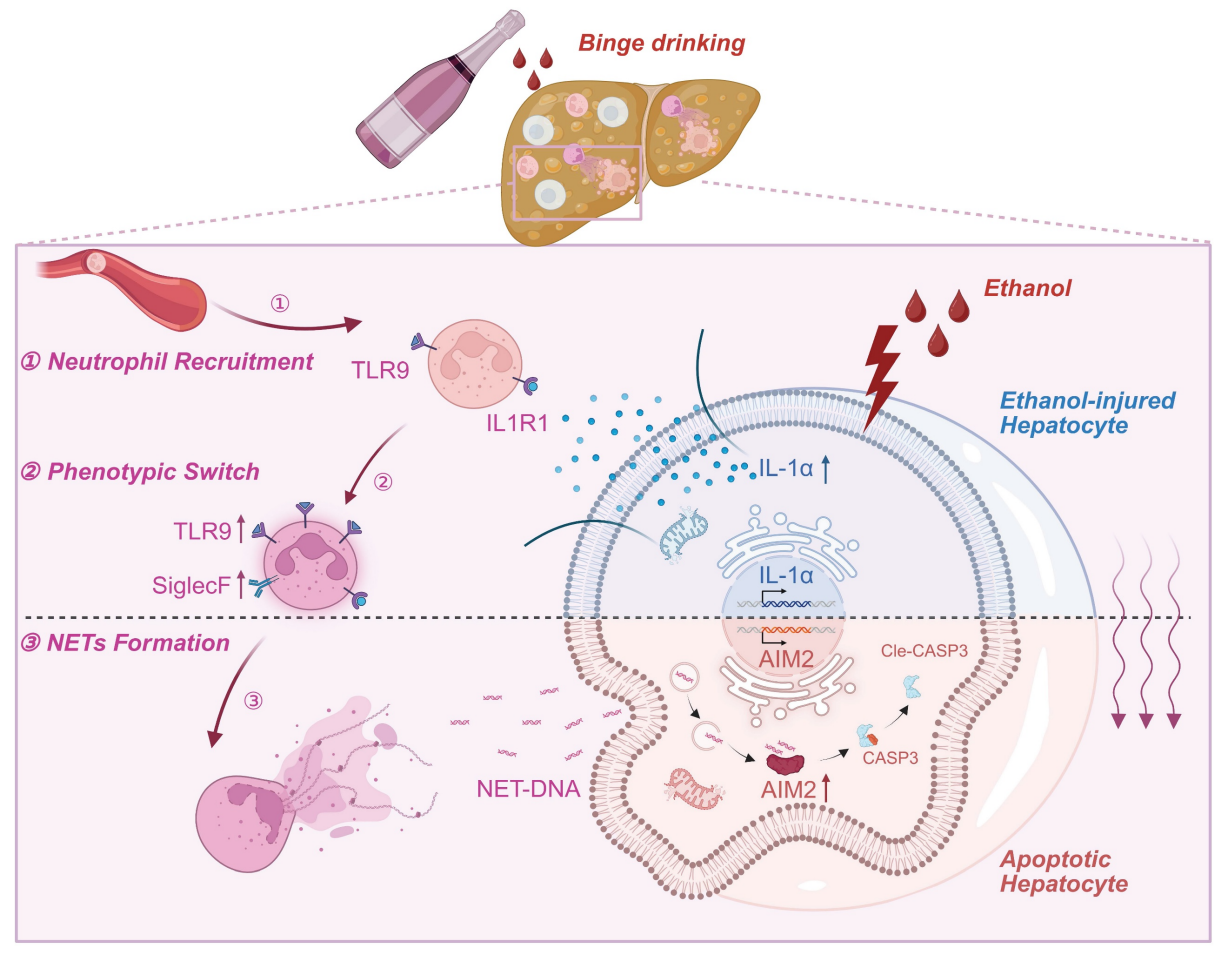
** Schematic model depicting the intercellular signaling axis between ethanol-injured hepatocytes and neutrophils during ASH progression.** Ethanol exposure triggers IL-1α release from hepatocytes, acting as a key damage-associated molecular pattern (DAMPs). This hepatocyte-derived IL-1α upregulates TLR9 expression in recruited neutrophils (①), thereby promoting a phenotypic switch toward a SiglecF⁺ NET-prone state (②). Enhanced NET formation follows (③), releasing extracellular NET-DNA, which is subsequently internalized by hepatocytes. Intracellular NET-DNA activates the cytosolic DNA sensor AIM2, leading to caspase-3 cleavage and hepatocyte apoptosis. This positive feedback loop sustains sterile hepatic inflammation and accelerates ASH pathogenesis.

ASH is widely recognized as a pivotal pathological stage in the progression of ALD, critically contributing to the development of cirrhosis and end-stage liver failure 26. In ASH, the liver is subjected to a dual assault comprising ethanol-induced cytotoxicity and sterile inflammatory responses 27. Hepatocytes, enriched in alcohol-metabolizing enzymes, are the first to sustain damage from ethanol metabolism 28. Upon injury, these hepatocytes release DAMPs into the circulation 29, which are sensed by immune cells that subsequently infiltrate the liver and initiate a robust sterile inflammatory cascade. Intense inflammatory response causes a secondary insult to hepatocytes, leading to massive hepatocyte death 30. The intermittent and recurrent activation of hepatic inflammation is a critical determinant in the transition toward liver fibrosis in ASH 4. Since hepatocytes and immune cells serve as the primary effectors of metabolic toxicity and sterile inflammation 31, respectively, elucidating the intercellular communication between these cell types is essential for advancing therapeutic strategies for ASH. In this study, we identified a previously unrecognized intercellular signaling axis between hepatocytes and neutrophils that drives liver injury in ASH. We demonstrated that ethanol-stressed hepatocytes release IL-1α as a key DAMP, which promotes TLR9 expression in neutrophils and enhances NETs formation. These NETs, in turn, activate the cytosolic DNA sensor AIM2 in hepatocytes, thereby triggering apoptosis (Figure 8). This feed-forward loop amplifies sterile hepatic inflammation and accelerates ASH progression.

Neutrophils are not only the most abundant leukocytes—accounting for approximately 70% of all white blood cells—but also the earliest immune cells recruited to sites of acute inflammation 32. Emerging evidence indicates that hepatic neutrophils actively participate in the regulation of hepatocyte death 33. Among their effector functions, NETs has been recognized as a key mechanism by which neutrophils execute immune responses 9. Moreover, NETs contribute to sterile inflammation and exacerbate tissue injury 12. Here, we provide compelling *in vivo* and *in vitro* evidence that NETs are major contributors to hepatocellular injury. We demonstrated that degradation of NETs by DNase I significantly attenuates hepatocyte apoptosis and reduces liver enzyme release. Furthermore, by establishing a sublethal hepatocyte injury model using ethanol, we show that NETs can transform otherwise non-lethal metabolic stress into a fatal apoptotic signal. These findings position NETs as potent amplifiers of liver damage, particularly within the metabolically impaired hepatic microenvironment of ASH.

Interleukins play essential roles in regulating neutrophil development, recruitment, activation, lifespan, and effector functions. For instance, IL-33 has been shown to exacerbate rhinovirus-induced asthma by promoting neutrophil recruitment and NET formation 34. Similarly, IL-19, secreted by renal tubular epithelial cells, functions as a key DAMP that induces neutrophil activation in aristolochic acid nephropathy 18. In our study, the observation that DNase I administration markedly reduced hepatocyte apoptosis suggests that the primary signal to neutrophils is likely transmitted by ethanol-injured hepatocytes, rather than by those undergoing cell death. Through screening the expression of 30 interleukins, IL-1α was identified as a key DAMP released by ethanol-injured hepatocytes. Functional assays revealed that IL-1α not only induces robust neutrophil chemotaxis but also potently enhances NET release. Mechanistically, transcriptomic profiling demonstrated that IL-1α acts as an upstream regulator of TLR9 expression in neutrophils. Prior studies have shown that TLR9 recognizes mtDNA and promotes NETosis 35; thus, IL-1α may synergize with mtDNA to drive neutrophil activation. Supporting this, pharmacological inhibition of TLR9 using E6446 significantly suppressed NET formation and attenuated hepatocyte apoptosis in ASH mice, underscoring its therapeutic potential. Notably, IL-1α also promoted the emergence of SiglecF⁺ neutrophils—a subset characterized by heightened NET-forming capacity 25—in a TLR9-dependent manner. This finding suggests that IL-1α/TLR9 signaling may induce phenotypic reprogramming of neutrophils, thereby contributing to the sustained pro-NETotic state observed in ASH.

NETs are complex web-like structures composed of dsDNA, histones, and neutrophil elastase, among other components 36. Upon stimulation with IL‑1α, NET formation is markedly enhanced, accompanied by substantial release of extracellular dsDNA. Consistently, we observed a significant increase in serum dsDNA levels in IL‑1α-treated mice (Figure S1B), confirming that IL‑1α promotes robust NET release *in vivo*. Excess extracellular dsDNA is a potent danger signal that can be recognized by cytosolic DNA sensors 37. Among these, NLRP3, cGAS, IFI16, and AIM2 have been reported to aggravate disease progression through recognition of NET-derived DNA in various conditions, including diabetic nephropathy 38, traumatic brain injury 39, and systemic lupus erythematosus 40. In our previous study, we demonstrated that in drug-induced liver injury, AIM2 can sense NET-DNA and trigger PANoptosis in hepatocytes 13. In this study, we found that AIM2 expression was also significantly upregulated in the ASH model, to a greater extent than other DNA sensors. However, unlike our previous findings in PANoptosis, ethanol-injured hepatocytes in the ASH model undergo classical apoptosis upon AIM2 activation. Notably, previous reports have shown that hepatocyte pyroptosis in ASH only occurs under conditions of severe inflammation 41, 42, suggesting that Binge feeding alone are insufficient to activate the full pyroptotic cascade. We further demonstrated that AIM2 colocalizes with intracellular NET-DNA in ethanol-injured hepatocytes. Genetic deletion of AIM2 protected against liver injury, whereas liver-specific AIM2 reconstitution restored NET sensitivity and hepatocellular apoptosis. Moreover, we confirmed that deletion of AIM2 did not alter the expression of CYP2E1, a key ethanol-metabolizing enzyme, indicating that the protective effects of AIM2 deficiency are not attributable to changes in alcohol metabolism 13. These findings reveal that AIM2 functions not only as a key mediator of hepatocyte responses to neutrophil-derived signals, but also as a critical executor of apoptosis in ethanol-injured hepatocytes.

A recent study has identified leukocyte cell-derived chemotaxin-2 (LECT2) as a potential signaling molecule mediating communication between hepatocytes and neutrophils 43. LECT2 was selected based on the expression of neutrophil chemokines and cell marker genes from both bulk and single-cell RNA sequencing datasets obtained from patients with ASH and healthy controls. It is postulated to act as a DAMP produced and secreted by hepatocytes under combined ethanol and immune-mediated stress. While LECT2 may indeed play a role in hepatocyte-neutrophil signaling, its temporal dynamics suggest that it is unlikely to function as an early-phase DAMP in ethanol-injured hepatocytes. In contrast, IL-1α was identified in our study as a DAMP released specifically from ethanol-injured hepatocytes. Beyond its chemotactic and activating effects on neutrophils, IL-1α also significantly upregulates the expression of TLR9, a receptor widely recognized as a key mediator of neutrophil activation 44, 45. Within the complex *in vivo* inflammatory microenvironment, IL-1α may synergize with mtDNA, a natural TLR9 ligand, to enhance neutrophil activation. These observations underscore the dual role of IL-1α in both the initiation and amplification of the inflammatory response. It is also worth discussing that AIM2, a cytosolic DNA sensor, raises important mechanistic questions about how extracellular NET-derived DNA gains access to the cytoplasm. Previous studies have reported that NET-DNA can be internalized by target cells through endocytosis 46. Additionally, hepatocytes are known to internalize a variety of extracellular substances via endocytic pathways 47. Using transmission election microscopy, we observed evidence of active endocytosis in hepatocytes from ASH model mice (Figure S5A), suggesting a possible route by which NET-DNA is internalized into ethanol-injured hepatocytes. Although our study confirmed that NET-DNA can be internalized by hepatocytes, the precise molecular mechanisms responsible for this process remain unclear. While broad-spectrum endocytosis inhibitors effectively blocked NET-DNA internalization, they also impaired the uptake of critical substances such as insulin 48 and iron 49, thereby disrupting hepatocyte homeostasis. This highlights the importance of future investigations into whether damaged hepatocytes possess selective recognition mechanisms for NET-DNA. Identifying specific pathways or receptors that mediate this uptake may provide novel strategies for protecting hepatocytes from NET-induced damage. This represents an intriguing and promising direction for further research.

Collectively, our findings support a model in which hepatocyte-derived IL-1α orchestrates a pathological feedback loop: it activates TLR9-expressing neutrophils, promotes NET formation, and in turn induces AIM2-dependent apoptosis in ethanol-stressed hepatocytes. Our work provides mechanistic insight into how sterile inflammation is initiated and amplified in ASH and identifies novel therapeutic targets for this life-threatening condition.

## Supplementary Material

Supplementary figures and table.

## Abbreviations

AAV8: Adeno-associated virus serotype 8
AIM2: Absent in melanoma 2
ASH: Alcohol-associated hepatitis
ALD: Alcohol-associated liver disease
ALT: Alanine aminotransferase
AML-12: Alpha mouse liver 12
AST: Aspartate aminotransferase
ASH: Alcohol-associated steatohepatitis
CitH3: Citrullinated histone H3
cGAS: Cyclic GMP-AMP synthase
DAMPs: Damage-associated molecular patterns
dsDNA: Double-stranded DNA
H&E: Hematoxylin and eosin
IFI16: Interferon gamma-inducible protein 16
IL: Interleukin
IL-1α: Interleukin-1 alpha
IHC: Immunohistochemistry
KO: Knockout
LDH: Lactate dehydrogenase
LECT2: Leukocyte cell-derived chemotaxin-2
MPO: Myeloperoxidase
mtDNA: Mitochondrial DNA
NETs: Neutrophil extracellular traps
NLRP3: NOD-, LRR- and pyrin domain-containing protein 3
PI: Propidium iodide
PMA: Phorbol 12-myristate 13-acetate
RNA-seq: RNA sequencing
scRNA-seq: Single-cell RNA sequencing
SiglecF⁺: Sialic acid-binding immunoglobulin-like lectin F positive
TLR9: Toll-like receptor 9
TUNEL: Terminal deoxynucleotidyl transferase dUTP nick end labeling
WT: Wild type
