# Supplementary material for: Neutrophil Recruitment via Hepatocyte IL-1α Drives NETs-Mediated AIM2 Hepatocyte Apoptosis in Alcohol-associated steatohepatitis
Source: Int J Biol Sci. 2025 Sep 3;21(13):5762–81. doi: 10.7150/ijbs.121255 (PMC12509912; doi:10.7150/ijbs.121255)
Supplement: Supplementary file 1 — Supplementary figures and table. [file ijbsv21p5762s1.pdf]

Supplementary figures

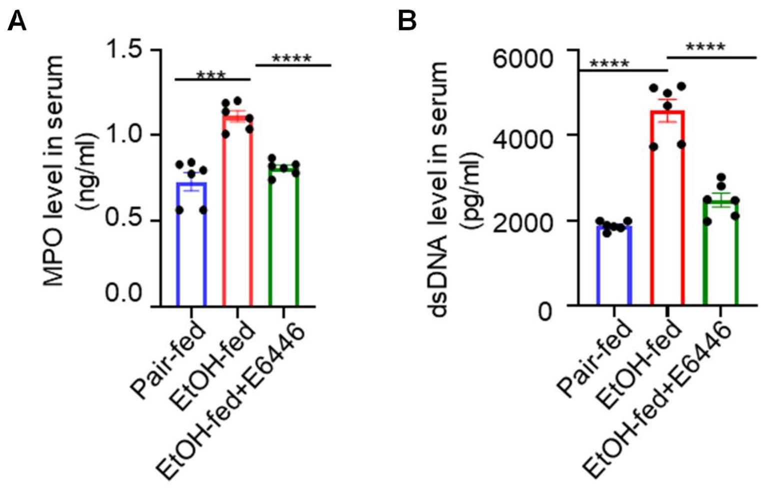

Figure S1. Pharmacological inhibition of TLR9 reduces NET formation in ASH mice.

(A, B) Serum levels of MPO (A) and dsDNA (B) were significantly elevated in EtOH-fed mice and reduced upon treatment with the TLR9 inhibitor E6446, indicating decreased NETs formation. Data are shown for pair-fed, EtOH-fed, and EtOH-fed + E6446 groups (n = 6 per group).

Data are presented as mean ± SEM. ****P*** < 0.001, *****P*** < 0.0001; ns, not significant. Statistical significance was determined using one-way ANOVA followed by Tukey's multiple comparisons test.

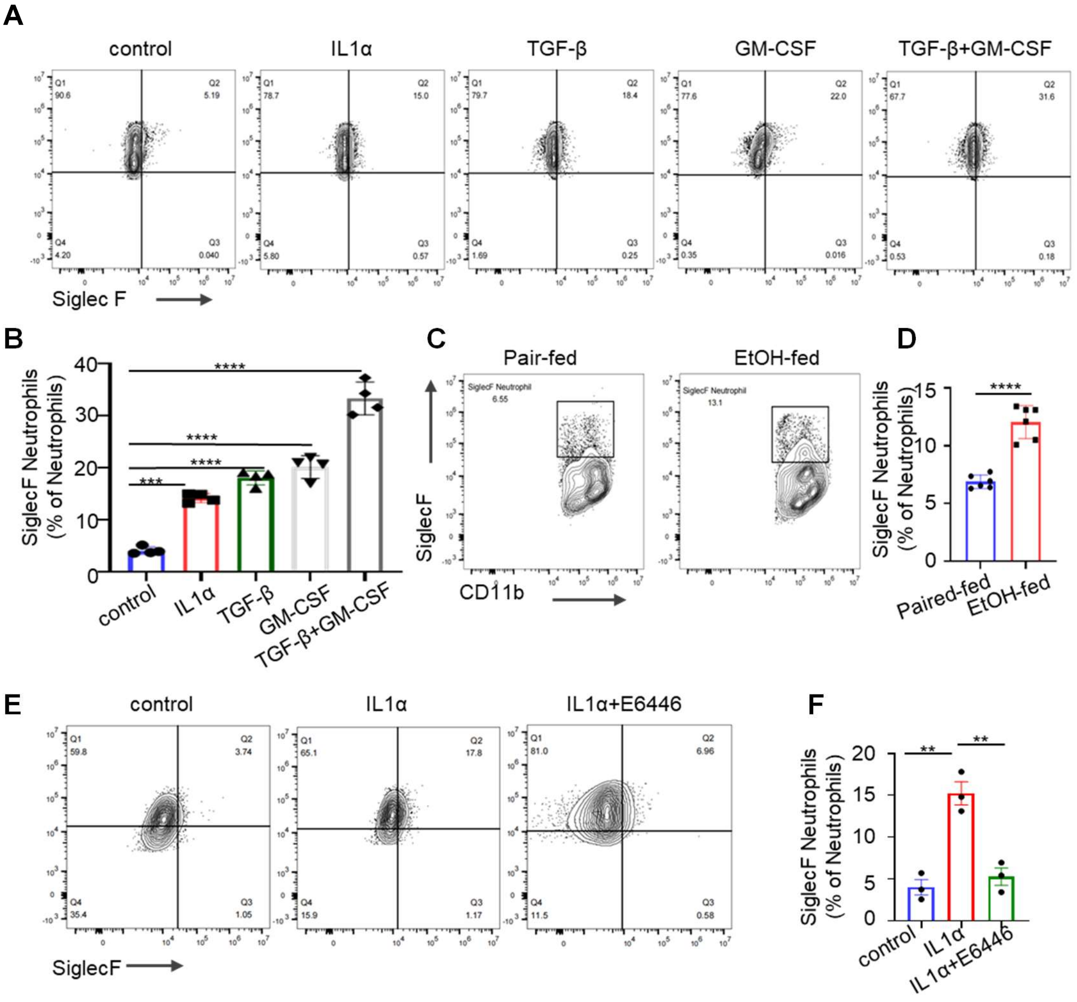

Figure S2. IL-1α induces SiglecF⁺ neutrophil phenotype in a TLR9-dependent manner.

(A) Representative flow cytometry plots showing the percentage of $SiglecF^+$ neutrophils within $CD11b^+Ly6G^+$ populations following 24 h stimulation with IL-1α (20 ng/mL), TGF-β (5 ng/mL), GM-CSF (10 ng/mL), or their combination.

(B) Quantification of $SiglecF^+$ neutrophils among total neutrophils across five treatment groups. IL-1α significantly upregulated SiglecF expression, albeit to a lesser extent than TGF-β or GM-CSF (n = 4 per group).

(C, D) Representative flow cytometry plots and quantification of $SiglecF^+$ neutrophils

in liver single-cell suspensions from pair-fed and EtOH-fed mice. The proportion of SiglecF$^+$ neutrophils was significantly higher in EtOH-fed mice (n = 6 per group).

(E, F) Flow cytometry plots and quantification showing that co-treatment with IL-1α and the TLR9 inhibitor E6446 markedly suppressed the IL-1α-induced SiglecF$^+$ neutrophil phenotype (n = 3 per group).

Data are presented as mean ± SEM. **P < 0.01, ***P < 0.001, ****P < 0.0001. Statistical analysis was determined using one-way ANOVA with Tukey's multiple comparisons test (B, F) or unpaired two-tailed t-test (D).

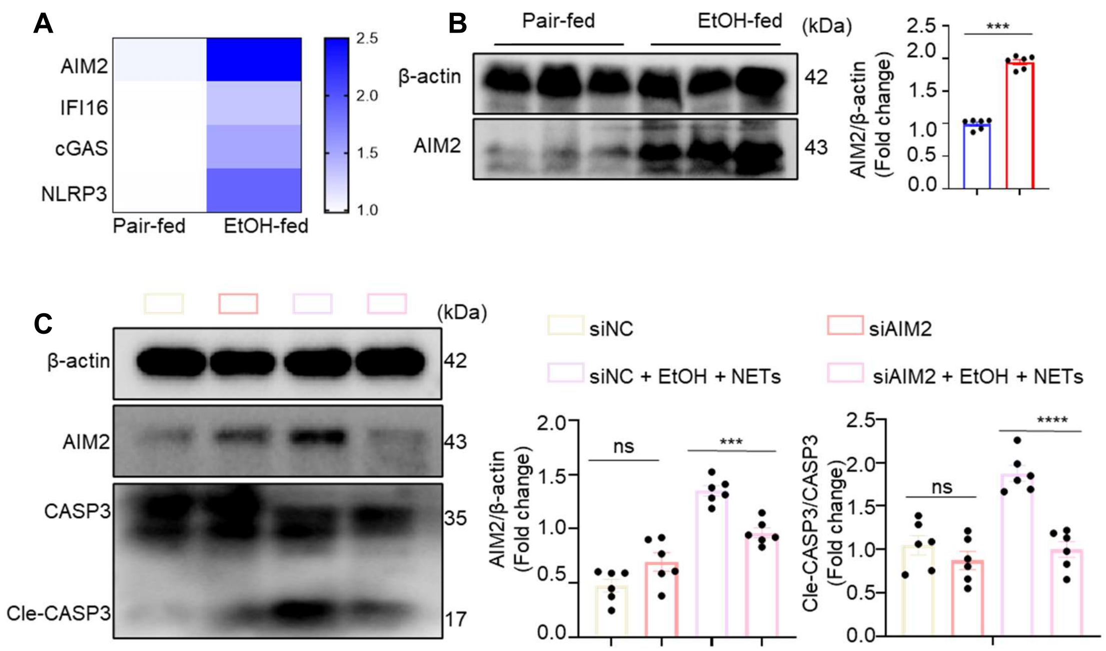

Figure S3. AIM2 is upregulated in ASH and mediates NET-induced hepatocyte apoptosis.

(A) Heatmap showing relative hepatic mRNA expression of dsDNA sensors (AIM2, IFI16, cGAS, and NLRP3) in pair-fed and EtOH-fed mice (n = 3 per group).

(B) Western blot and quantification of hepatic AIM2 expression in pair-fed and EtOH-fed mice confirmed significant protein upregulation (n = 6 per group).

(C) Western blot analysis of hepatocytes transfected with siRNA targeting AIM2 (siAIM2) or control siRNA (siNC), followed by stimulation with ethanol (100 µM, 12 h) and/or NETs (500 ng/mL). AIM2 knockdown attenuated NET-induced cleavage of caspase-3. Quantification of AIM2 expression (left) and the ratio of cleaved caspase-3 to total caspase-3 (right) is shown (n = 6 per group).

Data are presented as mean ± SEM. ***$\boldsymbol{P} < 0.001$, ****$\boldsymbol{P} < 0.0001$; ns, not significant. One-way ANOVA followed by Tukey's multiple comparisons test was used for statistical analysis.

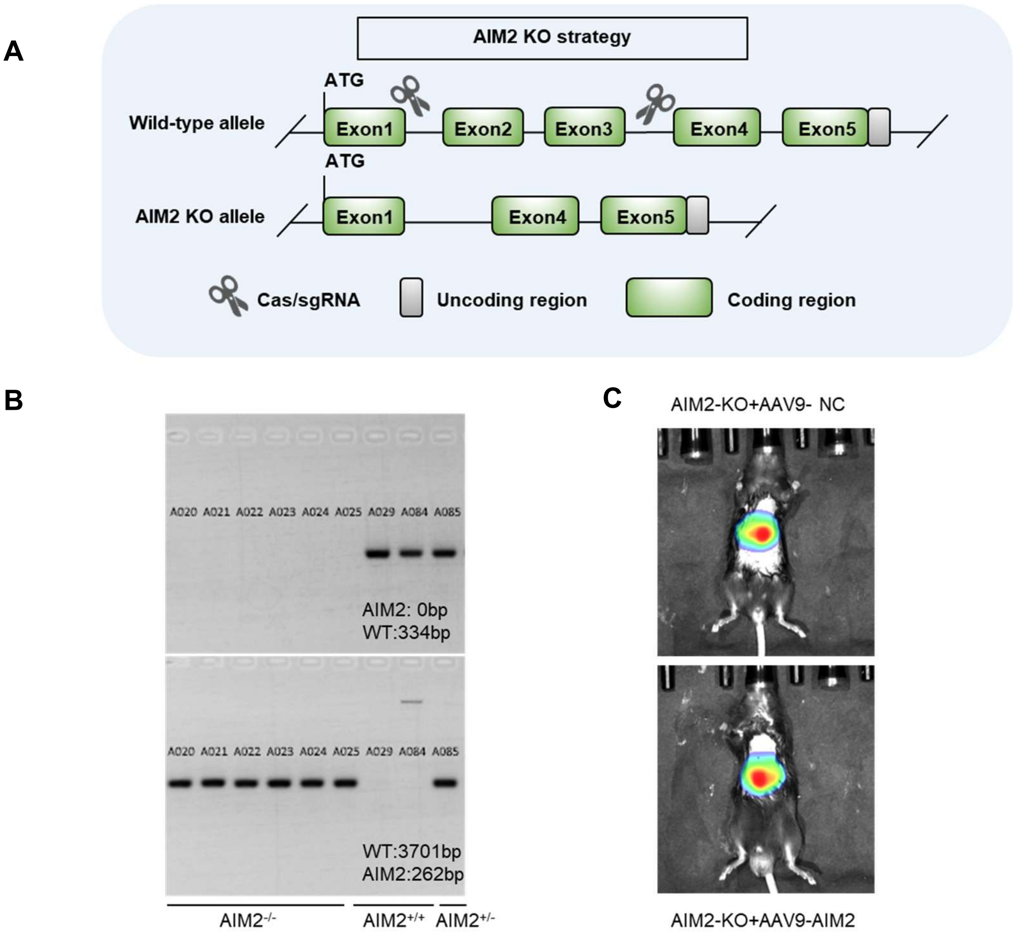

Figure S4. Generation and validation of AIM2-knockout mice and AAV-mediated hepatic AIM2 reconstitution.

(A) Schematic of the AIM2 gene editing strategy. Exons 2–3 of the Aim2-204 transcript (ENSMUST00000166137.2), corresponding to 566 bp of the coding region, were deleted using CRISPR/Cas9. sgRNAs targeting exon 2 and exon 3, along with Cas9 mRNA, were microinjected into fertilized C57BL/6JGpt embryos.

(B) Genotyping results of AIM2-knockout mice. Top: PCR amplification of the deleted region (WT: 334 bp; KO: 0 bp). Bottom: Long-fragment PCR validation (WT: 3701 bp; KO: 262 bp).

(C) Representative in vivo bioluminescent imaging of AIM2-KO mice injected via tail vein with AAV9-NC or AAV9-AIM2.

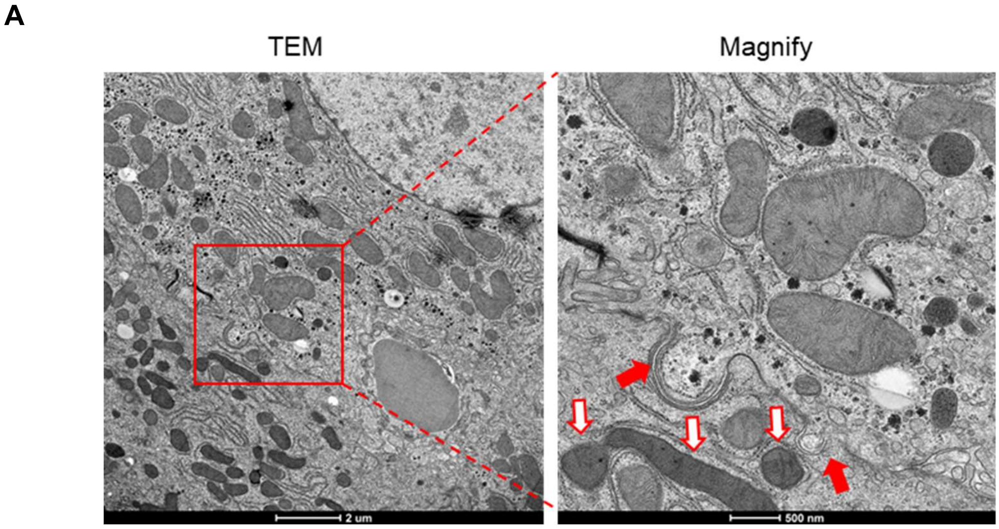

Figure S5. Transmission electron microscopy reveals endocytic vesicles in hepatocytes of ASH mice.

(A) Representative transmission electron microscopy (TEM) images of hepatocytes from ethanol-fed mice. The left panel shows a low-magnification overview of hepatic ultrastructure (scale bar = 2 µm); the boxed region is enlarged in the right panel (scale bar = 500 nm). Red arrows indicate intracellular vesicles undergoing endocytosis, suggesting active uptake of extracellular material, including NET-derived DNA. White arrows highlight hepatocyte mitochondria with condensed matrix and disrupted cristae, indicating mitochondrial damage. These features support the hypothesis that NET-derived DNA may enter hepatocytes through endocytic pathways under ethanol-induced stress.

Table 1. Primer sequences for the quantitative real-time PCR analysis of AML-12 cells and mouse tissues.

Terms (Mouse)	Forward primer (5'-3')	Reverse primer (5'-3')
IL1α	TCTATGATGCAAGCTATGGCTCA	CGGCTCTCCTTGAAGGTGA
IL1β	GAAATGCCACCTTTTGACAGTG	GAAATGCCACCTTTTGACAGTG
IL2	TGAGCAGGATGGAGAATTACAGG	TGAGCAGGATGGAGAATTACAGG
IL4	GGTCTCAACCCCCAGCTAGT	GCCGATGATCTCTCTCAAGTGAT
IL5	GCAATGAGACGATGAGGCTTC	GCCCCTGAAAGATTTCTCCAATG
IL7	TTCCTCCACTGATCCTTGTTCT	AGCAGCTTCCTTTGTATCATCAC
IL9	ATGTTGGTGACATACATCCTTGC	TGACGGTGGATCATCCTTCAG
IL10	CTTACTGACTGGCATGAGGATCA	GCAGCTCTAGGAGCATGTGG
IL12α	CAATCACGCTACCTCCTCTTTT	CAGCAGTGCAGGAATAATGTTTC
IL12β	GTCCTCAGAAGCTAACCATCTCC	CCAGAGCCTATGACTCCATGTC
IL13	TGAGCAACATCACACAAGACC	GGCCTTGCGGTTACAGAGG
IL14	TCCTGAGTACATACTGTGTGGAC	GCTGCATAGGTTCGGGACTTC
IL15	CATCCATCTCGTGCTACTTGTG	GCCTCTGTTTTAGGGAGACCT
IL16	AAGAGCCGGAAATCCACGAAA	GTGCGAGGTCTGGGATATTGC
IL17F	TGCTACTGTTGATGTTGGGAC	CAGAAATGCCCTGGTTTTGGT
IL17E	ACAGGGACTTGAATCGGGTC	TGGTAAAGTGGGACGGAGTTG
IL18	GTGAACCCCAGACCAGACTG	CCTGGAACACGTTTCTGAAAGA
IL19	CTCCTGGGCATGACGTTGATT	GCATGGCTCTCTTGATCTCGT
IL20	GTCTTGCCTTTGGACTGTTCT	AGGTTTGCAGTAATCACACAGC
IL21	GGACCCTTGTCTGTCTGGTAG	TGTGGAGCTGATAGAAGTTCAGG
IL22	ATGAGTTTTTCCCTTATGGGGAC	GCTGGAAGTTGGACACCTCAA
IL23	CAGCAGCTCTCTCGGAATCTC	TGGATACGGGGCACATTATTTTT

Terms (Mouse)	Forward primer (5'-3')	Reverse primer (5'-3')
IL24	GAGCCTGCCCAACTTTTTGTG	TGTGTTGAAGAAAGGGCCAGT
IL27	CTGTTGCTGCTACCCTTGCTT	CTCCTGGCAATCGAGATTCAG
IL28B	GTTCAAGTCTCTGTCCCCAAAA	GTGGGAACTGCACCTCATGT
IL31	GTTCAAGTCTCTGTCCCCAAAA	TCGCTCAACACTTTGACTTTCT
IL33	ATTTCCCCGGCAAAGTTCAG	AACGGAGTCTCATGCAGTAGA
IL34	TTGCTGTAAACAAAGCCCCAT	CCGAGACAAAGGGTACACATTT
IL40	ACTGGAAGTTTATCCCCAAAGC	CGGAGTCATGCACAACCTTTTT
Aim2	GTCACCAGTTCCTCAGTTGTG	CACCTCCATTGTCCCTGTTTTAT
IFI16	AAAGGAGCCTGCTAAGGAAGA	CGTTCACATCAGAGACACAGGA
cGAS	CACGAAGCCAAGACGCCCTC	GTCGCACTTCAGTCTTCCCTTTTT
NLRP3	ATTACCCGCCCGAGAAAGG	TCGCAGCAAAGATCCACACAG
GAPDH	GTCTTCACTACCATGGAGAAGG	TCATGGATGACCTTGGCCAG
